# Efficacy of different types of cognitive enhancers for patients with schizophrenia: a meta-analysis

**DOI:** 10.1038/s41537-018-0064-6

**Published:** 2018-10-25

**Authors:** Igne Sinkeviciute, Marieke Begemann, Merel Prikken, Bob Oranje, Erik Johnsen, Wan U. Lei, Kenneth Hugdahl, Rune A. Kroken, Carina Rau, Jolien D. Jacobs, Silvia Mattaroccia, Iris E. Sommer

**Affiliations:** 1 Division of Psychiatry, Haukeland University Hospital, PB 1400, 5021 Bergen, Norway; 2 NORMENT Centre of Excellence, University of Oslo, Oslo, Norway; 3 Centre for Research and Education in Forensic Psychiatry, Haukeland University Hospital, Bergen, Norway; 4 Rijks Universiteit Groningen (RUG), University Medical Center Groningen, Department of Neuroscience and Department of Psychiatry, Groningen, The Netherlands; 5 Brain Center Rudolf Magnus, University Medical Center Utrecht, Utrecht, The Netherlands; 6 Department of Clinical Medicine, Section of Psychiatry, Faculty of Medicine, University of Bergen, Bergen, Norway; 7 School of Medicine, Medical Sciences and Nutrition, University of Aberdeen, Aberdeen, UK; 8 Department of Biological and Medical Psychology, University of Bergen, Bergen, Norway; 9 Department of Chemistry, University of Konstanz, Konstanz, Germany; 10 Department Graduate School of Life Science, Faculty of Sciences, Utrecht University, Utrecht, The Netherlands; 11 Department of Dynamic and Clinical Psychology, Faculty of Medicine and Psychology, Sapienza University of Rome, Rome, Italy

## Abstract

Cognitive impairment is a core feature of schizophrenia, which is predictive for functional outcomes and is, therefore, a treatment target in itself. Yet, literature on efficacy of different pharmaco-therapeutic options is inconsistent. This quantitative review provides an overview of studies that investigated potential cognitive enhancers in schizophrenia. We included pharmacological agents, which target different neurotransmitter systems and evaluated their efficacy on overall cognitive functioning and seven separate cognitive domains. In total, 93 studies with 5630 patients were included. Cognitive enhancers, when combined across all different neurotransmitter systems, which act on a large number of different mechanisms, showed a significant (yet small) positive effect size of 0.10 (*k* = 51, *p* = 0.023; 95% CI = 0.01 to 0.18) on overall cognition. Cognitive enhancers were not superior to placebo for separate cognitive domains. When analyzing each neurotransmitter system separately, agents acting predominantly on the glutamatergic system showed a small significant effect on overall cognition (*k* = 29, Hedges’ *g* *=* 0.19, *p* = 0.01), as well as on working memory (*k* = 20, Hedges’ *g* = 0.13, *p* = 0.04). A sub-analysis of cholinesterase inhibitors (ChEI) showed a small effect on working memory (*k* = 6, Hedges’ *g* = 0.26, *p* = 0.03). Other sub-analyses were positively nonsignificant, which may partly be due to the low number of studies we could include per neurotransmitter system. Overall, this meta-analysis showed few favorable effects of cognitive enhancers for patients with schizophrenia, partly due to lack of power. There is a lack of studies involving agents acting on other than glutamatergic and cholinergic systems, especially of those targeting the dopaminergic system.

## Introduction

Cognitive impairment is a core feature of schizophrenia.^1^ A range of cognitive functions are affected in patients with schizophrenia, with a mean decrease of one to two standard deviations (SD) compared to the general population.^2^ Cognitive dysfunction can be present before the onset of psychotic symptoms and after the first psychosis either remains at decreased levels or declines further during the illness.^3^ These deficits are predictive for functional outcomes, both before and after the first psychotic episode^4,5^ and are, therefore, a treatment target of its own.^6^ Psychological as well as pharmacological treatments have been suggested for cognitive enhancement in schizophrenia. Cognitive remediation techniques have been investigated in depth and have shown small to medium effects, irrespective of active or passive control groups.^7^ Antipsychotic medication predominantly target positive symptoms such as delusions, hallucinations, and disorganization, and may even have a negative impact on cognition.^8^

The pathophysiology of cognitive dysfunctions in schizophrenia is complex and many different neurotransmission systems are involved.^9^ Therefore, pharmacological agents targeting different putative mechanisms may be relevant for cognitive enhancement. Pharmacological enhancement of cognition has been a main field of research in the last decades, investigating different neurotransmitter systems and seemingly reporting as many positive as negative findings. As of yet, there is no clear picture whether any pharmacological treatment can improve cognitive functioning in schizophrenia, directly or indirectly by increasing the efficacy of other treatment modalities, and whether or not further investigation of specific cognitive enhancers, i.e., pharmacological agents targeting neurotransmitter systems that are potentially relevant for improving cognitive impairment, should be encouraged.^9^

Several meta-analyses and reviews have been conducted regarding potential cognitive enhancers. One of the most extensive ones was conducted by Choi et al.^10^ where the authors reviewed agents acting on three different neurotransmitter systems, including glutamatergic, cholinergic, and serotonergic. However, the majority of systematic reviews either includes one specific neurotransmitter system only, or do not perform meta-analytical calculations. We here aim to provide a comprehensive overview of current literature on cognitive enhancers for schizophrenia as well as answer the question if and which cognitive enhancers improve overall cognition or one of the specific cognitive domains. We summarize the efficacy of cognitive enhancers acting on seven different neurotransmitter systems (including a miscellaneous group), thereby making a distinction between the different neurotransmitter systems targeted. Even though the cognitive enhancers act on very different (and sometimes opposite) brain mechanisms, we also perform one overarching analysis including all different categories, as all drugs have the same aim, namely: to improve cognition. We only included published, high quality, double-blind studies that compared an enhancer to placebo.

For the discussion, we used the arbitrary cutoff values of effect sizes > 0.02 as clinically significant and total samples of *n* < 1000 for an underpowered area of research.

## Results

The outcome of literature search is shown in Supplementary Material S1, demographic information on all included studies is provided in Table 1. In total, 93 studies with 5630 patients were suitable for inclusion in the meta-analysis (glutamatergic: *k* = 27; cholinergic: *k* = 32; serotonergic: *k* = 14; dopaminergic: *k* = 3; GABA-ergic: *k* = 2; noradrenergic: *k* = 4; miscellaneous: *k* = 11) (see Table 1). The mean sample size per study sample was 28.73 (SD = 27.13, range = 4–203), mean age of the participants was 44.15 years (SD = 6.36, as reported by 91 study samples), 68.54% of the sample were men (as reported by 87 study samples) and average illness duration was 15.57 years (SD = 6.47, as reported by 63 study samples). The cognitive domains covered in the included studies are shown in Supplementary Material S2.

**Table 1:** Characteristics of included studies and overview of putative cognitive-enhancing agents

Study	*N*	Age (years)^a^	Gender (M/F)	Illness duration (years)^b^	Cognitive enhancer	Other specified psychotropic medication	Dosage (mg/day)	Treatment duration (days)^c^
Glutamatergic system								
Tsai, 1998^43^	CE: 14 P: 15	33.9 ± 6.6 31.7 ± 7.5	6/8 10/5	10.7 ± 6.7 10.5 ± 6.0	d-serine	Antipsychotics	30	42
Goff, 1999^44^	CE: 15 P: 22	46.8 ± 12.3 41.2 ± 8.1	15/8 19/4	22.3 ± 13.3 18.5 ± 8.6	d-cycloserine	FGA	50	56
Tsai, 1999^45^	CE: 10 P: 10	42.6 ± 3.6 39.5 ± 5.5	NR	20.6 ± 6.1 19.9 ± 5.7	d-serine	Clozapine	30	42
Goff, 2001^46^	CE: 12, P:6	39.8 ± 10.5	16/3	19.8 ± 5.6	Ampakine CX516	Clozapine	3600	28
Duncan, 2004^47^	CE: 7 P: 8	48.7 ± 12.1 54.4 ± 11.8	10/0 10/0	25.8 28.6	d-cycloserine	FGA, benztropine, trihexyphenidyl, propanolol	50	28
Silver, 2005^48^	CO: 23	36.9 ± 10.8	22/7	10.5 ± 8.8	Amantadine	FGA, SGA, SSRIs, anticholinergic medication, benzodiazepines, carbamazepine	200	21 (4th week)^d^
Buchanan, 2007^49^	CE: 37 CE: 40 P: 38	42.6 ± 10.8 44.4 ± 10.4 43.4 ± 11.4	NR	20.2 ± 10.0 21.8 ± 11.1 20.2 ± 11.0	Glycine d-cycloserine	Antipsychotics except clozapine, anticholinergics, beta-blockers, mood stabilizers, antidepressants, antianxiety or anticonvulsant medications	60000 50	112
Zoccali, 2007^50^	CE: 26 P: 25	32.5 ± 6.9 30.2 ± 7.8	15/11 13/12	9.3 ± 3.3 10.4 ± 4.3	Lamotigrine	Clozapine, lorazepam	200	168
Goff, 2008a^51^	CE: 45 P: 49	42.0 ± 9.3 43.7 ± 11.0	44/7 43/11	NR	Ampakine CX516	Clozapine, olanzapine, risperidone	2700	28
Goff, 2008b^52^	CE: 16 P: 16	50.1 ± 9.15 48.0 ± 6.66	10/9 13/6	23.9 ± 12.5 21.6 ± 8.7	d-cycloserine	Antipsychotics except clozapine	50mg^h^	56
de Lucena, 2009^53^	CE:10 P:11	34.6 ± 10.0 34.7 ± 8.6	8/2 11/11	18.6 ± 8.6 17.2 ± 8.3	Memantine	Clozapine, benzodiazepines	20	84
Lieberman, 2009^54^	CE: 61 P: 62	40.9 ± 9.8 40.1 ± 11.3	41/28 53/14	16.6 ± 9.6 16.4 ± 10.6	Memantine	SGA, mood Stabilizers, SSRIs, venlafaxine, mirtazapine	20	56
Marx, 2009^55^	CE: 9 P: 9	52.7 ± 6.3 49.4 ± 12.2	8/1 9/0	NR	Pregnenlone	SGA, antidepressants, mood stabilizers, anticholinergics	500	56
Levkovitz, 2010 ^56^	CE: 13 P: 8	25.1 ± 4.8 24.7 ± 4.2	25/11 15/3	NR	Minocycline	SGA	200	154
Chengappa, 2012^57^	CE: 30 P: 29	46.6 ± 8.5 46.5 ± 9.0	21/12 23/14	NR	L-carnosine	Antipsychotics, anticholinergics, mood stabilizers	2000	90
Lee, 2012^58^	CE: 15 P:11	44.3 ± 4.3 43.4 ± 3.9	11/4 5/6	14.3 ± 8.6 11.3 ± 11.3	Memantine	FGA, anti-parkinsonian anticholinergics, benzodiazepines	20	84
Weiser, 2012^59^	CE: 69 P: 64	39.4 ± 12.0 39.8 ± 12.3	74/23 70/28	17.1 ± 11.7 15.3 ± 10.3	d-serine	Antipsychotics, anticholinergic agents, beta-blockers, mood stabilizers, anxiolytics, antidepressants	2000	112
Vayisoglu, 2012^60^	CE: 16 P: 17	40.5 ± 9.9 41.2 ± 10.9	10/7 13/4	17.8 ± 7 18.7 ± 10.7	Lamotigrine	Clozapine	200	84
D’Souza, 2013^61^	CE: 27, P: 26	37.2	78/26	10.7	d-serine	Antipsychotics, anticholinergic agents, benzodiazepines	30	84
Lane, 2013^62^	CE: 23 P: 26	38.4 ± 9.7 36.3 ± 7.9	11/14 15/12	16.2 12.9	Benzoate	Antipsychotics	1000	42
Liu, 2014^63^	CE: 39 P: 40	27.1 ± 5.7 27.7 ± 7.3	25/14 24/16	1.8 ± 1.2 2.3 ± 1.2	Minocycline	Risperidone, alprazolam, trihexyphenidyl hydrochloride, propranolol	200	112
Schoemaker, 2014^64^	4–8 mg: 62 12–16 mg: 67 P: 62	37.4 ± 9.5 38.8 ± 11.0 38.1 ± 10.5	41/30 46/27 46/24	NR	Org 25935	SGA other than clozapine	8–16 24–32	56
Kelly, 2015^65^	CE: 27 P: 23	42.9 ± 14.2 42.3 ± 11.0	20/8 18/5	24.4 23.0	Minocycline	Clozapine	200	70
Lin, 2015^66^	Sarcosine: 21 Sarcosine + Benzoate: 21 P: 21	38.2 ± 9.3 37.8 ± 9.6 39.1 ± 9.5	15/6 11/10 13/8	14.7 ± 6.6 12.6 ± 7.3 13.8 ± 8.5	Sarcosine Sarcosine + Benzoate	Antipsychotics	2000 2000 + 1000	84
Veerman, 2016^67^	CO: 52	42.4 ± 9.6	39/13	22.9 ± 8.0	Memantine	Clozapine	20	84 (15th week)^d^
Kantrowitz, 2017^68^	CO: 14	40.0 ± 11.0	13/1	23.0 ± 12.0	d-serine	Antipsychotics other than clozapine	60	42 (9th week)^d^
Mazinani, 2017^69^	CE: 23 P: 23	44.8 ± 6.6 45.3 ± 6.2	23/0 23/0	23.5 ± 8.3 25.7 ± 5.4	Memantine	Risperidone	20	84
Cholinergic, system								
Friedman, 2002^70^	5 mg: 10, 10 mg: 8 P: 18	50.3 ± 10.1 48.8 ± 11.1	16/2 16/2	26.9 ± 9.6 25.9 ± 13.9	Donepezil	Risperidone, benzodiazepines	5 10	84
Smith, 2002^11^	CO: 29	40.8 ± 6.9	30/1	24.2 ± 6.5	Nicotine ^e^	Antipsychotics, lithium, valproic acid, benztropine mesylate, trihexyphenidyl	10	Challenge
Tugal, 2004^d^^71^	CE: 6 P: 6	29.2 ± 5.9 38.0 ± 10.2	4/2 2/4	6.3 ± 3.1 16.0 ± 9.0	Donepezil	FGA	5	42
Kumari, 2006^72^	CE: 11 P: 10	42.6 ± 8.8 44.4 ± 11.6	9/2 5/5	17.5 15.6	Rivastigmine^e^	SGA	12	84
Schubert, 2006^73^	CE: 8 P: 8	48.3 ± 6.9 46.8 ± 8.8	16/1	NR	Galantamine^f^	Risperidone	24	56
Smith, 2006^12^	CO: 25	37.6 ± 8.3	26/0	NR	Nicotine	Antipsychotics, mood stabilizers	10	Challenge
Fagerlund, 2007^74^	CE: 7 P: 4	33.2 ± 7.6 35.0 ± 6.2	4/3 4/0	6.7 ± 5.2 8.1 ± 5.9	Donepezil	Ziprasidone, SSRI, SNRI, chlorprotixene, zopiclone, benzodiazepines	10	120
Kohler, 2007^75^	CE: 11 P: 11	31.7 ± 8.0 30.1 ± 6.2	7/4 9/2	< 10	Donepezil	SGA	10	112
Lee, 2007^76^	CE: 11 P: 12	42.2 ± 5.7 44.2 ± 4.0	8/4 7/5	13.1 ± 4.7 15.9 ± 5.7	Donepezil	Haloperidol, anti-parkinsonian anticholinergics, benzodiazepines	5	84
Lee, 2007^77^	CE: 12 P: 12	39.5 ± 3.2 41.5 ± 3.2	8/4 6/6	15.8 ± 5.7 18.8 ± 7.2	Galantamine	FGA, anti-parkinsonian anticholinergics, benzodiazepines	16	84
Akhondzadeh, 2008^78^	CE: 15 P: 15	32.3 ± 6.5 33.9 ± 6.1	9/6 10/5	7.1 ± 3.9 7.4 ± 4.2	Donepezil	Risperidone	10	84
Barr, 2008^13^	CO: 28	47.0 ± 8.0	16/12	NR	Nicotine^g^	Antipsychotics	14	Challenge
Buchanan, 2008^79^	CE: 35 P: 38	49.9 ± 9.2 49.5 ± 9.9	37/5 37/7	25.5 23.6	Galantamine	SGA other than clozapine, FGA	24	84
Dyer, 2008^80^	CE: 10 P: 10	44.3 ± 11.9 50.5 ± 4.7	7/3 6/4	NR	Galantamine^g^	Antipsychotics	32	56
Freedman, 2008^81^	CE: 31, P: 31	22 - 60	22/9	NR	DMXB-A	FGA, SGA	150 300	28
Keefe, 2008^82^	CE: 111 P: 115	40.9 ± 9.7 39.7 ± 9	87/34 85/39	18.0 14.8	Donepezil	SGA	10	84
Shiina, 2010 ^83^	CE: 16 P: 17	35.0 ± 6.8 35.2 ± 8.5	9/11 10/10	12.0 ± 8.7 9.8 ± 6.4	Tropisetron	Risperidone, anticholinergics, benzodiazepines, lithium, milnacipran and trazodone, valproic acid, carbamazepine	10	56
Hong, 2011^84^	CE: 32 P: 32	44.0 ± 1.8 41.6 ± 1.9	20/12 22/10	NR	Varenicline	FGA, SGA	1	56
Lindenmayer, 2011^85^	CE: 15 P: 17	41.9 ± 10.8 38.5 ± 12.2	10/5 12/5	19.0 15.9	Galantamine	Risperidone	24	180
Velligan, 2012^86^	5 mg: 88 20 mg: 74 35/100 mg: 65 P: 86	41.7 ± 7.8 40.9 ± 8.4 40.6 ± 9.2 40.6 ± 9.3	80/36 64/28 69/28 72/33	16.8 ± 9.1 15.6 ± 9.6 15.4 ± 9.3 16.3 ± 9.5	AZD3480	SGA	5 20 35/100	84
Shim, 2012^87^	CE: 46 P: 45	39.9 ± 8.6 39.9 ± 9.9	38/21 45/13	13.5 ± 7.8 14.2 ± 9.9	Varenicline	Antipsychotics, lorazepam, anticholinergic medications	2	56
Zhang, 2012^31^	5 mg: 8 10 mg: 10 20 mg: 10 P: 10	29.6 ± 8.9 27.1 ± 5.9 31.5 ± 9.9 33.6 ± 9.8	8/2 7/3 8/2 7/3	4.1 ± 3.2 4.2 ± 3.9 3.4 ± 4.0 3.5 ± 4.4	Tropisetron	Risperidone, chloral hydrate	5 10 20	10
Deutsch, 2013^88^	CE: 19, P: 24	53.3 ± 9.9	39/4	26.0	(Galantamine + CDP-choline)	SGA	24 + 2000	112
Lieberman, 2013^89^	CE: 76 P: 78	36.3 36.3	65/29 63/28	NR	TC-5619	Quetiapine, risperidone	25	84
Umbricht, 2014^90^	5 mg: 47 15 mg: 50 50 mg: 48 P: 49	40.1 ± 8.3 39.6 ± 9.6 40.5 ± 8.9 38.1 ± 9.9	35/19 37/16 37/17 40/14	NR	RG3487	Antipsychotics	5 15 50	56
Zhu, 2014^91^	CE: 26 P: 26	24.7 ± 5.9 25.9 ± 4.4	14/17 11/19	4.8 ± 2.5 5.1 ± 2.5	Donepezil	Risperidone, olanzapine	5	84
Keefe, 2015^92^	0.27 mg: 54 0.9 mg: 55 P: 57	39.1 ± 9.7 37.3 ± 10.5 39.2 ± 9.9	70/37 75/30 70/35	≥ 3	Encenicline	SGA other than clozapine, SSRI	0.27 0.9	84
Walling, 2015^93^	5 mg: 100 50 mg: 101 P: 203	40.0 38.4 38.6	81/40 75/46 141/94	NR	TC-5619	SGA other than clozapine, sertindole and melperone	5 50	168
Haig, 2016a^94^	10 mg: 54 25 mg: 63 P: 65	42.0 ± 9.0 41.0 ± 10.0 44.0 ± 9.0	43/26 40/27 48/19	17.0 ± 11.0 15.0 ± 9.0 18.0 ± 9.0	ABT-126	SGA, some anxiolytics and hypnotics	10 25	84
Haig, 2016b^95^	CE: 134 P: 122	40.1 ± 12.1 42.4 ± 11.4	79/72 81/63	≥ 2	ABT-126^g^	Antipsychotics, anticholinergic agents	50	168
Shoja Shafti and Azizi Khoei 2016^96^	CE: 18 P: 18	44.6 ± 5.8 46.4 ± 4.1	18/0 18/0	23.6 ± 6.2 23.9 ± 5.5	Rivastigmine	FGA	12	84
Buchanan, 2017^97^	CE: 15 P: 15	45.8 ± 12.4 42.2 ± 11.7	14/6 17/3	NR	Galantamine	Antipsychotics	12	42
Serotoninergic system								
Sumiyoshi, 2001^98^	CE: 15 P: 11	27.8 ± 6.3 31.8 ± 9.4	9/6 6/5	6.3 ± 4.3 7.5 ± 5.4	Tandospirone	FGA, biperiden	30	42
Poyurovsky, 2003^99^	CE: 11 P: 13	42.5 ± 12.9 45.5 ± 7.5	8/3 9/4	15.4 ± 11.9 18.7 ± 11.7	Mianserin	FGA, benzodiazepines, anticholinergic agents, valproic acid	15	28
Friedman, 2005^100^	CO: 19	44.9 ± 7.1	NR	NR	Citalopram	SGA	40	168 (12th week)^d^
Sumiyoshi, 2007^101^	CE: 30 P: 29	41.6 ± 12.7 41.6 ± 11.7	16/14 16/13	19.4 ± 14.6 19.5 ± 11.2	Buspirone	SGA	30	180
Akhondzadeh, 2009^102^	CE:15 P: 15	33.0 ± 5.9 33.5 ± 6.0	10/5 9/6	7.1 ± 3.4 7.3 ± 4.0	Ondansetron	Risperidone	8	84
Berk, 2009^103^	CE: 18 P: 20	37.8 ± 10.9 35.9 ± 9.2	14/4 18/2	NR	Mirtazapine	SGA, benzodiazepines	30	42
Piskulic, 2009^104^	CE: 9 P: 9	43.4 ± 10.3 37.2 ± 13.7	6/3 8/1	15.2 ± 10.2 11.7 ± 9.4	Buspirone	SGA	15	42
Stenberg, 2010 ^105^	CE: 19 P: 18	44.1 ± 9 48.1 ± 10	10/9 9/9	20.2 ± 9.3 24.4 ± 9.5	Mirtazapine	FGA	30	42
Mico, 2011^106^	CE: 20 P: 20	35.9 ± 7.1 34.0 ± 6.8	11/9 13/7	6.8 ± 3.1 6.1 ± 3.2	Duloxetine (SNRI)	Clozapine	60	112
Morozova, 2012^107^	CE: 21 P: 20	34.6 ± 10.5 35.8 ± 14.1	21/0 20/0	12.1 ± 8.7 8.6 ± 7.5	Dimebon / Latrepirdine	Risperidone	20	28
Niitsu, 2012^108^	CE: 23 P: 24	38.6 ± 9.5 36.3 ± 9.4	14/9 15/9	12.3 ± 9.3 10.8 ± 7.5	Fluvoxamine	SGA, anticholinergic mediation, mood stabilizers, tranquilizers	50	28
Morozova, 2014^109^	CE: 17 P: 25	34.9 ± 10.0 37.1 ± 1.8	NR	13.9 17.5	AVN-211	Antipsychotics	4	28
Sheikhmoonesi, 2015^110^	CE: 25 P: 25	46.7 ± 9.5 47.3 ± 10.6	20/5 20/5	≥ 2	Buspirone	FGA	30	42
Samadi, 2017^111^	CE: 18 P: 20	36.6 40.4	16/2 19/1	NR	Ondansetron	Risperidone	8	84
Dopaminergic system								
Pietrzak, 2010 ^112^	CO: 32	43.3	32/0	13.2 ± 10.2	d-amphetamine	SGA	10	Challenge
Kaphzan, 2014^113^	CE: 23 P: 22	41.8 ± 2.7 43.8 ± 2.3	17/6 16/6	10.8 ± 2.8 11.0 ± 4.0	Entacapone	Antipsychotics	600	84
Girgis, 2016^114^	0.5 mg: 16 15 mg: 16 P: 17	38.6 ± 8.8 39.5 ± 9.2 40.4 ± 9.5	8/8 9/7 8/9	NR	DAR-0100A	Antipsychotics	0.5 15	5
GABA-ergic system								
Lewis, 2008^115^	CE: 9 P: 6	39.3 ± 10.6 34.0 ± 3.6	9/0 6/0	15.2 ± 10.8 16.4 ± 10.7	MK-0777	Antipsychotics	16	28
Buchanan, 2011^116^	6 mg: 15 16 mg: 18 P: 17	43.3 ± 9.3 44.9 ± 8.7 40.0 ± 10.9	11/7 13/8 16/5	NR	MK-0777	SGA other than clozapine	6 16	28
Noradrenergic system								
Friedman, 2001^117^	CE: 19 P: 19	49.1 ± 11.0 47.3 ± 10.4	16/3 16/4	26.2 ± 13.0 24.4 ± 13.2	Guanfacine	FGA, risperidone	2	28
Friedman, 2008^118^	CE: 7 P: 8	NR	NR	NR	Atomoxetine	SGA	80	56
Kelly, 2009^119^	CE: 10 P: 12	48.9 ± 5.7 49.1 ± 8.5	8/2 8/4	NR	Atomoxetine	SGA other than clozapine and aripiprazole, benztropine, lorazepam, other psychotropic medications (except venlafaxine, monoamine oxidase, other anticholinergics and benzodiazepines)	80	56
Poyurovsky, 2009^120^	CE: 16 P: 17	33.5 ± 10.6 28.8 ± 7.6	10/6 11/6	4.2 ± 5.5 3.0 ± 3.5	Reboxetine (NRI)	Olanzapine, anticholinergics, benzodiazepines	4	42
Miscellaneous								
Sevy, 2005^121^	CE: 10 P: 10	35.9 ± 9.4 38.9 ± 10.0	5/5 3/7	11.6 ± 9.0 12.8 ± 10.8	Modafinil	FGA, SGA, anticholinergic agents, mood stabilizers, benzodiazepines, antidepressants, buspirone, zolpidem	200	56
Pierre, 2007^122^	CE: 10 P: 10	49.7 ± 6.8 49.8 ± 7.0	10/0 9/1	NR	Modafinil	SGA	200	56
Goff, 2009^123^	CO: 17	49.7 ± 0.6	NR	NR	Sildenafil (PDE Inhibitors)	Psychiatric medications	50 100	Challenge (48 h)
Kane, 2010 ^124^	50 mg: 14 100 mg: 14 150 mg: 12 P: 13	44.9 ± 10.9 40.4 ± 9.6 41.4 ± 9.8 46.0 ± 7.8	11/4 10/5 11/4 12/3	NR	Armodafinil	Risperidone, olanzapine, paliperidone	50 100 150	28
Bobo, 2011^125^	CE: 29 P: 29	44.0 ± 14.6 38.8 ± 11.7	15/14 20/9	22.9 ± 15.5 17.5 ± 11.1	Armodafinil	Antipsychotics, SSRIs	150	42
Javitt, 2012^126^	5 mg: 17 30 mg: 18 P: 19	43.2 ± 10.5 45.2 ± 8.2 41.4 ± 10.4	41/22 41/22 41/22	NR	Davunetide AL-108	FGA, SGA, lithium	5 30	84
Kane, 2012^127^	150 mg: 70 200 mg: 69 250 mg: 71 P: 70	43.7 ± 11.2 43.1 ± 11.1 44.4 ± 9.4 42.4 ± 10.1	53/18 57/13 50/22 46/26	18.6 ± 11.3 16.7 ± 9.9 17.5 ± 10.6 17.7 ± 11.2	Armodafinil	Risperidone, olanzapine, paliperidone	150 200 250	168
Yi, 2012^128^	CE: 9 P: 10	41.4 ± 10.3 39.7 ± 7.5	7/2 7/3	NR	Rosiglitazone	Clozapine	4	56
Lohr, 2013^129^	CE: 12 P: 12	47.8 ± 13.0 48.5 ± 8.8	12/0 12/0	14.5 ± 9.7 19.8 ± 8.5	Modafinil	SGA, antidepressant, anticholinergic, benzodiazepine, anticonvulsant	50–200	56
Huerta-Ramos, 2014^130^	CE: 14 P: 12	60.1 ± 6.4 62.7 ± 4.5	0/16 0/17	27.7 ± 7.0 25.2 ± 11.1	Raloxifene	Antipsychotics, biperiden, benzodiazepines, antidepressants	60	84
Lees, 2017^131^	CO: 40	25.6 ± 4.9	30/10	21.5 ± 9.4	Modafinil	SGA other than clozapine	200	Challenge

*CE* cognitive enhancers group, *P* placebo group, *CO* crossover study, *FGA* first-generation antipsychotics, *SGA* second-generation antipsychotics, *SSRIs* selective serotonin reuptake inhibitors, *SNRIs* serotonin–norepinephrine reuptake inhibitors, *NRI* norepinephrine reuptake inhibitor, *NR* not reported

^a^Mean of age is reported, if not range

^b^Mean of illness duration calculated from onset age and mean age if not provided

^c^Data at first phase of the crossover study is used, i.e., before crossover;

^d^Point of crossover that could follow 2 weeks of ‘‘washout’’

^e^Nicotine smokers

^f^Nicotine smokers, except one

^g^Non-smokers

^h^Once-weekely dosing–50 mg per week

### Overall analyses combining cognitive enhancers from different neurotransmitter systems

**Fig. 1:**
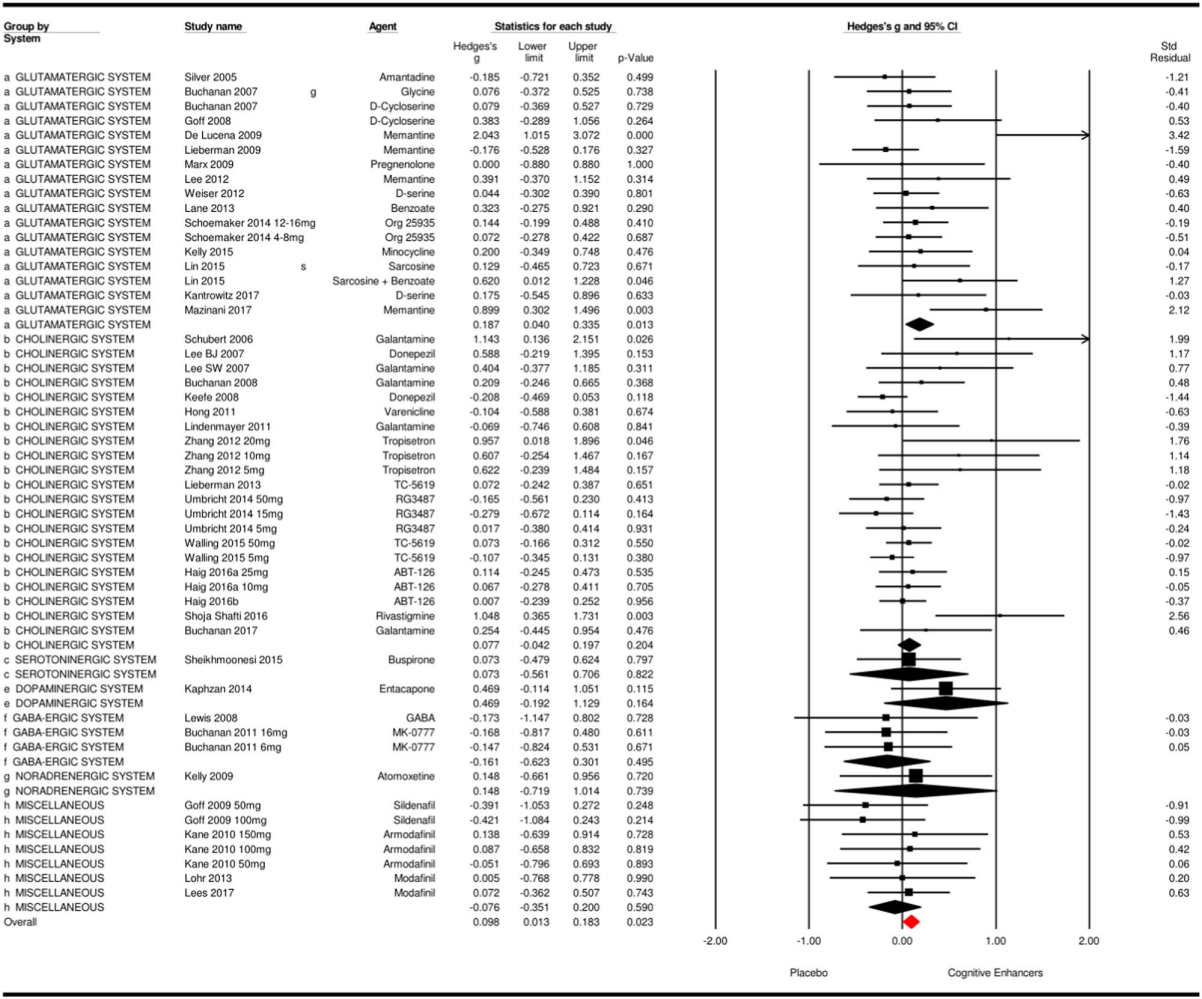
Effects of cognitive enhancers on overall cognitive functioning

Combining all cognitive enhancers across different neurotransmitter systems for the efficacy on overall cognition resulted in 51 study samples, with a total of 3635 patients (see Fig. 1). Cognitive enhancers showed a very small but significant positive effect size of 0.10 over placebo treatment (*p* = 0.023; 95% CI = 0.01 to 0.18). The number of missing null studies to render this positive result to nonsignificance was 105. The significant *Q*-value (*Q*(50) = 70.84, *p* *=* 0.028) showed that the variability among studies was higher than would be expected due to sampling error, and further examination of subgroup differences is warranted. Heterogeneity was low to moderate (*I*^*2*^ = 29.4%), indicating that 29% of the dispersion that reflects differences in the true effect sizes, while the remaining 71% can be attributed to random sampling error. The funnel plot and Egger’s test (*t* = 3.95, *p* < 0.001) indicated potential publication bias (see Supplementary Material 3).

In spite of their very small, yet significant effect on overall cognition, taken as a group, cognitive enhancers had no significant positive effect on any of the separate cognitive domains (see Table 2). Furthermore, meta-regression analyses showed no associations between the effect of cognitive enhancers on overall cognition and duration of treatment (*z* = −0.09, *p* = 0.929) or illness duration (*z* = 0.67, *p* = 0.505). The effect sizes for the cognitive subdomains also showed no relation between duration of treatment or illness duration (14 regressions, *p*-values ranging between 0.107 and 0.927).

**Table 2:** Effects of all combined cognitive enhancers on separate cognitive domains

Cognitive domain	Number of studies (*k*)	Patients (*N*)	ES Hedges’s *g*	(95% CI)	*p*-value	Q-statistic (df)	*I* ^2^	Failsafe *N*_R_
Overall cognition	51	3635	0.10	(0.01 to 0.18)	0.023	*Q*(50) = 70.84,*p* = 0.028	29.41	105
Attention	71	4435	0.01	(0.07 to 0.08)	0.893	*Q*(70) = 97.73,*p* = 0.016	28.38	0
PS	71	4782	0.01	(0.04 to 0.07)	0.647	*Q*(70) = 50.33,*p* *=* 0.963	0.00	0
Reasoning	74	4492	0.02	(−0.05 to 0.08)	0.639	*Q*(76) = 90.38,*p* = 0.082	19.22	0
Verbal fluency	27	1134	−0.05	(−0.16 to 0.07)	0.400	*Q*(26) = 22.71,*p* = 0.649	0.000	0
Verbal L&M	74	4190	0.03	(−0.03 to 0.10)	0.327	*Q*(73) = 82.75,*p* *=* 0.204	11.79	0
Visual L&M	66	4133	0.05	(−0.02 to 0.11)	0.155	*Q*(65) = 67.04,*p* *=* 0.407	3.04	0
WM	80	4649	0.06	(−0.01 to 0.12)	0.069	*Q*(79) = 93.58,*p* *=* 0.125	15.58	0

#### Efficacy of cognitive enhancers targeting the glutamatergic system

Twenty-nine study samples of agents acting predominantly on the glutamatergic system were grouped into this category. Seventeen study samples evaluated the effects on overall cognition, showing a small but significant effect of 0.19 (*p* = 0.013) (see Fig. 1). The significant *Q*-value indicated that the included studies did not share the same effect size (*Q*(16) = 28.2, *p* = 0.030), while heterogeneity was low to moderate (*I*^*2*^ = 43.30%). The funnel plot and Egger’s test (*t* = 3.03, *p* < 0.001) indicated potential publication bias (see Supplementary Material 4). Furthermore, a very small effect was found for these agents on working memory, as compared to placebo (*k* = 20, Hedges’ *g* = 0.13, *p* = 0.040) (see Supplementary Material S5 and S6).

Further sub-analyses were performed by grouping agents acting mainly at the glycine site (sarcosine, benzoate, glycine, d-serine, and d-cycloserine, *k* = 12 in total), agents targeting the AMPA receptor (*k* = 5) and memantine/amantadine agents (*k* = 6), (see Supplementary material S5). Glycine site acting agents did not show superior effects compared to placebo, which may result from insufficient statistical power. AMPA receptor agonists were more effective than placebo in improving working memory (*k* = 5, Hedges’ *g* = 0.28, *p* = 0.030). However, the *Q*-statistic was significant and heterogeneity was moderate to large (*Q*(3) = 8.911, *p* = 0.030; *I*^*2*^ = 66.33%), although no outliers were identified. Furthermore, the effects of memantine/amantadine on overall cognition showed a positive trend (*k* = 6, Hedges’ *g* = 0.34, *p* = 0.063), which could reach significance when power increases.

#### Efficacy of cognitive enhancers targeting the cholinergic system

Forty-three study samples targeting the cholinergic system could be included. Meta-analysis did not show superior effects of these agents compared to placebo, (see Fig. 1 and Supplementary Material S5 and S6). When excluding challenge studies (i.e., studies providing only a single dose^11–13^), results did not change. Sub-analyses were performed showing no superior effects for nicotinergic agents (three to twenty studies per domain). When subdividing into alpha 7 (three to sixteen studies per domain) and alpha 4 (zero to five studies per domain) nicotinic agonists, no significant results were found except for alpha 4 nicotinic agonists showing a more favorable outcome for placebo in problem solving (*k* = 5, Hedges’*g* *=* −0.175 and *p* = 0.027). The cholinesterase inhibitors (ChEI) showed a small yet significant effect on working memory (*k* = 6, Hedges’ *g* = 0.26, *p* = 0.031), while no significant effects were found in the sub-analyses for galantamine (two to six studies per domain).

#### Efficacy of cognitive enhancers targeting the serotoninergic system

Fourteen study samples evaluated cognitive enhancers that target the serotonergic system. The efficacy of these agents was not superior to placebo (see Fig. 1 and Supplementary Material S5 and S6). When performing sub-analyses for (partial) 5-HT1A agonists (zero to three studies per domain), or antidepressants (zero to five studies per domain), neither comparison reached significance.

#### Efficacy of cognitive enhancers targeting the dopaminergic system

For agents targeting the dopamine system, only four study samples could be included. Studies could only be combined for the domain of reasoning, the positive effect size did not reach significance (*k* = 4, Hedges’ *g* = 0.34, *p* = .072), which could be caused by insufficient statistical power. For overall cognition, and the domains of attention, processing speed, and visual learning and memory, only single studies were available so no mean weighed effect size could be calculated (individual effect sizes are noted in Supplementary Material S5).

#### Efficacy of cognitive enhancers targeting the GABA-ergic system

Three study samples could be included in the GABA-ergic system. Mean weighed effect sizes were nonsignificant for this type of cognitive enhancers as compared to placebo (see Fig. 1 and Supplementary Material S5 and S6).

#### Efficacy of cognitive enhancers targeting the noradrenergic system

Four study samples were included targeting the noradrenergic system, showing no significant effects on overall cognition or the separate cognitive domains (see Fig. 1 and Supplementary Material S5 and S6).

#### Efficacy of cognitive enhancers targeting miscellaneous receptor systems

Seventeen study samples were included in this category. The effects of these cognitive enhancers compared to placebo were nonsignificant (see Fig. 1 and Supplementary Material S5 and S6). However, when excluding challenge studies, the placebo group showed a superior effect on attention (*k* = 12, Hedges’ *g* = −0.16, *p* = 0.038; *I*^*2*^ = 0%).

## Discussion

Cognitive dysfunction is a key problem in schizophrenia that largely defines global functioning. Therefore, interventions to improve cognition are needed urgently. Here, we quantitatively summarized literature on 93 studies investigating the efficacy of pharmacological treatment for cognitive impairment in schizophrenia. We reviewed the efficacy of agents acting on seven categories of different neurotransmitter systems, evaluating overall cognition as well as seven cognitive subdomains.

### All pharmacological agents combined

The results for cognitive enhancers of all neurotransmitter systems taken together on overall cognition showed a significant effect. Although statistical significance was reached, the small size of the effect prevents a positive recommendation for their clinical use as of yet, as the small improvement is easily outweighed by the risk of side-effects. When specific cognitive domains were analyzed, the effects were close to zero, which indicates that very small advantages are to be expected of augmentation with an enhancer.

### Efficacy of glutamatergic cognitive enhancers

The glutamatergic system is one of the most investigated systems in enhancing cognition in schizophrenia. Previous meta-analyses conducted on agents acting on the glutamatergic system by Tsai et al.^14^ showed positive results on cognition measured by the PANSS cognitive subscale (ES = 0.28, *p* = 0.002), whereas a meta-analysis by Choi et al.^10^ did not find any effect of included agents, neither on overall cognition nor on cognitive subdomains. Another meta-analysis by Iwata et al.^15^ did not find superiority of glutamate positive modulators over placebo. Although they reported that AMPA receptor positive modulators did have a tendency to improve attention/vigilance, this finding did not survive statistical corrections. All three meta-analyses differed in terms of included compounds, number of studies and subjects, which might explain different results. Our meta-analysis included 13 different compounds in 27 studies with a total of 1540 patients. Thus, our meta-analysis is the largest and includes the most agents acting on the glutamatergic system compared to previous ones. Overall, we concluded that glutamatergic agents provide some beneficial effects on overall cognition and working memory, but with questionable clinical importance, given the small effect sizes of 0.19 for overall cognition and 0.13 for working memory. Sub-analyses indicated that agents acting on the AMPA site provided larger effects on working memory (ES = 0.28). In addition, memantine/amantadine might also be promising, as the medium effect size for overall cognition bordered on significance. However, more studies on these specific agents are needed for final conclusions as these sub-analyses included less than 1000 individuals and heterogeneity between the studies was indicated.

### Efficacy of cholinergic cognitive enhancers

Several meta-analysis and reviews have been conducted for the cholinergic system. A recent meta-analysis by Kishi et al.^16^ found no significant differences between the effects of antipsychotics plus add-on anti-dementia drugs or add-on placebo on either overall cognition or cognitive subdomains. However, the meta-analysis by Kishi et al. combined cholinesterase inhibitors and glutamatergic antagonists (memantine), which makes comparisons to our sub-analysis complicated. Lewis et al.^17^ conducted a meta-analyses of alpha-7 nicotinic agents in neuropsychiatric disorders, were the majority of studies included schizophrenia patients. The authors found very modest beneficial effects, where only a sub-analysis of a subgroup with the most effective doses reached significance on the overall cognitive index (ES = 0.13, *p* = 0.02). In our meta-analysis, seven studies had two or three intervention groups with different doses. We included all investigated doses, which might explain differences in the results. In the meta-analysis by Choi et al.^10^ 13 studies investigating cholinesterase inhibitors (ChIE) were included. The authors found a trend for a positive effect of ChEI on verbal learning and memory. Although the number of included studies for verbal learning and memory are the same, we did not replicate their results for this domain. The differences of methods between Choi et al.^10^ and our meta-analysis could explain this disparity in results. The review of pharmaceutical cognitive-enhancing agents in schizophrenia and bipolar disorder by Vreeker et al.^18^ described galantamine as promising for schizophrenia. We found a trend for significant results in processing speed.

In conclusion, some small beneficial effects for cognition might be achieved by ChEI, especially in the working memory domain, although the meta-analysis show moderate heterogeneity and a modest sample size (*n* = 364). More research is needed on the effects of galantamine on cognition as the effect size for several domains were above 0.2 but the meta-analysis for this drug was also underpowered.

### Efficacy of serotonergic cognitive enhancers

For the serotonergic system, a previous meta-analysis by Choi et al.,^10^ including five studies of 5HT_1A_ receptor agonists and one study of 5HT_2A_ antagonist mianserin, did not find any favorable effects of these agents. However, a previous meta-analysis by Vernon et al.^19^ on antidepressants for cognitive impairment in schizophrenia included 11 studies with agents acting predominantly on the serotonergic system and found a small effect on composite cognition score as well as a small effect on executive function (ES = 0.10, *p* = 0.01 and ES = 0.17, *p* = 0.02, respectively). In line with Vernon et al.,^19^ we found no positive effects on other analyzed domains. In conclusion, very small effect sizes and lack of statistical significance indicate that targeting the serotonergic system alone might not result in sufficient cognitive enhancement in patients with schizophrenia, with the possible exception of the attention domain (ES = 0.23).

### Efficacy of dopaminergic cognitive enhancers

Too few studies were suitable for inclusion to investigate dopaminergic substances as a venue to improve other than reasoning cognitive domains and, therefore, no conclusions can be made at this point. This is very unfortunate, as the dopaminergic system, especially the frontal D_1_ system is thought to be central to the cognitive dysfunction seen in schizophrenia.^20^ Thus, further research on dopaminergic enhancers such as methylphenidate, is needed urgently.

### Efficacy of GABA-ergic cognitive enhancers

For the GABA-ergic system the investigated agent is described as being selective for GABA_A_ α2 and α3 receptors subunits and should not be sedating, however, this effect cannot be completely excluded and could therefore have influenced our results. GABA hypofunction is thought to underlie at least part of the cognitive impairment seen in schizophrenia.^21^ Results from our meta-analysis should be considered with much caution as only five domains with eighty two patients in each were analyzed.

### Efficacy of noradrenergic cognitive enhancers

Both norepinephrine reuptake inhibitors (atomoxetine, reboxetine) and stimulation of α_2A_ receptors (guanfacine, clonidine) or blockade of α_2C_ or α_1_ receptors have been suggested as putative mechanisms for cognitive enhancement.^22,23^ However, individual studies included in our meta-analysis, found no beneficial effects of norepinephrine reuptake inhibitors (atomoxetine, reboxetine), whereas guanfacine showed some efficacy. Results from our meta-analysis are underpowered for this neurotransmitter system, as only three domains with seventy to seventy five patients per domain were analyzed.

### Efficacy of cognitive enhancers of the miscellaneous category

No beneficial effects for the miscellaneous group or modafinil/armodafinil subgroup were found. Recent reviews on modafinil and armodafinil^24^ and modafinil alone^25^ reported beneficial effects in single dose studies for some cognitive domains, in particular working memory. All, except one of the reviewed studies with longer treatment duration found no beneficial effects of modafinil/armodafinil on cognition. Our results are based on longer treatment duration studies and indicate no beneficial effects of modafinil/armodafinil for cognition in patients with schizophrenia in a longer time frame.

### Limitations

Combining pharmacological agents across different neurotransmitter systems increases heterogeneity among studies; however multiple neurotransmitter systems are involved in cognitive dysfunction in schizophrenia and all these different classes of drugs have the same aim (i.e., to improve cognition). Therefore, we believe that a combined overall effect does provide valuable information about cognitive enhancers as a group. Most included studies investigating cognitive enhancers are relatively small and generally include older individuals in their chronic phase of illness, while effects may be better in an earlier stage. Furthermore, most studies provide experimental treatment of short duration and use performance-based measures of cognition. While older chronic patients may not benefit from the drugs targeting cognitive functions, short duration of treatment might not be enough for the changes to be clinically visible. The choice of cognitive measurements and selection of composite scores for overall cognitive functioning might lack sensitivity to detect subtle changes. If we had also included subjective measures of cognition (for example, using the PANSS item on cognition), effect sizes may have been larger, but the results could also have been more difficult to interpret.

Grouping of the different agents according to their presumed mechanism of action is rather challenging as some of them have several different putative receptor targets (for example tropisetron, mirtazapine, modafinil) or the mechanisms are not well known. Yet, given the large diversity in cognitive enhancers that has been studied in schizophrenia, some grouping is necessary to draw any meaningful conclusions, as few compounds have been used in multiple studies. Accordingly, our strategy was to start the analysis with broad categories based on the putative predominant neurotransmitter systems involved. However, as some of the potential enhancers act very differently on the same neurotransmitter system (e.g., the glutamate category include both glycine site NMDA receptor agonists and NMDA receptor antagonists), we broke the analyses further down into more selective categories whenever sample sizes permit.

Another possible limitation is our inclusion of single dose studies. Although not similar to treatment studies, we think that these studies do provide valuable data. Nevertheless, for those analyses where these were included, we performed a sensitivity analysis after excluding single dose studies.

Different cognitive tests are used in the studies. Issues concerning test batteries, such as practice effects, ceiling or floor effects, placebo effects,^26^ or sensitivity of the test may have influenced the results. However, these are well known issues and suggestions for future studies are provided elsewhere.^26^

Finally, this meta-analysis focused on schizophrenia, schizophreniform and schizoaffective disorder. Schizotypal personality disorder (SPD) was not included as it is categorized among the personality disorders in DSM5. SPD does however share some of the cognitive deficits seen in patients with schizophrenia, although to a lesser extent. Indeed, several studies of cognitive enhancers in patients with SPD have shown positive results.^27,28^ If SPD had been included in the present meta-analysis the combined effect sizes might accordingly become somewhat larger.

### Directions for future research

Given the limitations mentioned above, future studies should consider the following recommendations:

*1. Using the optimal dose*

Some of the agents have a very narrow therapeutic window, where too much or too little does not improve cognition but may in fact worsen it (e.g., agents stimulating D_1_R^29,30^), while for other agents the optimal therapeutic dose is still uncertain.^17,31^ For substances such as d-serine, tropisetron, reboxetine, modafinil, armodafinil dose finding in small groups is the first necessary step before large scale RCTs should be started.

*2. Including young patients*

Most of the studies include chronic patients in a stable phase of their illness. As chronic patients are usually older, brain plasticity is likely to be more limited. Since plasticity is highly associated with cognitive functions, future studies should include a younger population, specifically when investigating systems mediating neuroplasticity, such as the GABAergic and glutamatergic system.

*3. Treat at least 6 months*

MATRICS recommends phase III clinical trials of at least 6 month duration to be able to determine efficacy and endurance.^32^ However, most of the trials are significantly shorter. Thus, negative findings may stem from insufficient duration of treatment.

*4. Investigating more homogeneous groups*

Patients with schizophrenia display a heterogeneous clinical picture that is likely to reflect different pathologies at the brain level. Therefore drugs might have differential effects for subgroups of patients. Disentangling the different pathologies underlying cognitive deficiencies should be a target to better stratify the different cognitive enhancers in subgroups of patients that share some common “biomarkers”. Such biomarkers could be detected with EEG coherence measures (for the GABA system), with Event Related Potentials on EEG (for example for the noradrenergic system) or with the Short Latency Afferent Inhibition test (a combination of TMS over the motor cortex and an EMG read out, a reflection of cholinergic innervation).^33^

Taken as a group, we found a significant (small) effect of cognitive enhancers in patients with schizophrenia. For specific agents, few positive results emerged. Yet, enhancers acting on the glutamatergic system showed a small positive effect on overall cognition and working memory, while treatment with ChIE had a significant positive effect on working memory, albeit with a small effect size. Results favoring placebo might represent chance findings, yet the possibility that alpha4 agents, ChIE and GABAergic agents might actually worsen some cognitive functions cannot be rejected. There is still a major lack of reports on agents acting on other systems, especially the dopaminergic and noradrenergic systems. Important issues such as dose, treatment duration, including a younger population and subtyping heterogeneous samples should be taken into consideration when planning future studies.

## Methods

### Neurotransmitter systems

In our systematic search we included different pharmacological agents, targeting the following neurotransmitter systems:

(i) The glutamatergic system: glycine, d-serine, d-cycloserine, CX516, amantadine, memantine, pregnenolone, minocycline, l-carnosine, lamotrigine, benzoate, Org 25935, sarcosine;
(ii) The cholinergic system: nicotine, donepezil, rivastigmine, galantamine, DMXB-A, tropisetron, varenicline, AZD3480, TC-5619, ABT-126;
(iii) The serotonergic system: tandospirone, mianserin, mirtazapine, citalopram, buspirone, ondansetron, duloxetine, latrepirdine, fluvoxamine, ANV-211;
(iv) The dopaminergic system: d-amphetamine, entacapone, DAR-0100A;
(v) The GABA-ergic system: MK-0777;
(vi) The noradrenergic system: atomoxetine, reboxetine, guanfacine;
(vii) Miscellaneous-including agents that do not specifically target the aforementioned neurotransmitter systems, or that target multiple systems, or for which the exact target system is not well known: davunetide, rosiglitazone, raloxifene, sildenafil, armodafinil, modafinil.

### Literature search

The meta-analysis was performed according to the Preferred Reporting for Systematic Reviews and Meta-analysis (PRISMA) Statement.^34^ A systematic search for studies published in peer-reviewed journals was conducted in PubMed (Medline), Psychinfo, EmBase and Cochrane Database of Systematic Reviews.

Combinations of the following search terms were used: ‘‘schizophrenia’’, ‘‘schizoaffective’’, ‘‘schizophreniform’’, ‘‘psychosis’’, ‘‘cognition’’, ‘‘cognitive’’, ‘‘enhancers’’, ‘‘enhancement’’, ‘‘glutamatergic’’, ‘‘glutamate’’, ‘‘NMDA’’, ‘‘AMPA’’, ‘‘cholinergic’’, ‘‘acetylcholine’’, ‘‘acetylcholinesterase’’, ‘‘nicotinergic’’, ‘‘muscarinergic’’, ‘‘serotonin’’, ‘‘serotonergic’’, ‘‘dopamine’’, ‘‘dopaminergic’’ ‘‘D1’’, ‘‘COMT’’, ‘‘noradrenaline’’, ‘‘noradrenergic’’, ‘‘GABA-ergic’’, ‘‘GABA’’, and the individual names of cognitive enhancers as mentioned in 2.1, with no year or language limits. The literature search was conducted by three authors (C.R., J.D.J., and S.M.), where at least two of them searched independently for relevant publications. Titles, abstracts, and then relevant full-text papers were examined. Consensus was reached between the authors in cases of discrepancy. Cross references from the relevant papers were searched for additional publications. If necessary, corresponding authors were contacted to provide details needed for study inclusion in the meta-analyses.

Inclusion/exclusion criteria

#### Inclusion criteria

1. Randomized, placebo-controlled studies measuring the effect of pharmacological agents on cognition.
2. Studies including patients with schizophrenia, schizophreniform, schizoaffective, delusional, or psychotic disorder not otherwise specified according to the diagnostic criteria of the Diagnostic and Statistical Manual of mental Disorders (DSM-III[-R], DSM-IV[R], DSM-V), or the International Classification of Diseases (ICD-9 or -10).
3. Cognitive functioning is measured with neuropsychological tests.
4. Studies reporting sufficient information to compute common effect size (ES) statistics (i.e., means, mean changes, SDs, exact *F*-, *p*-, *t*-, or *z*-values) or corresponding authors provided these data upon request.

#### Exclusion criteria

1. Studies evaluating cognition solely based on more subjective measures, such as an item from the PANSS (Positive and Negative Syndrome Scale)^35^ interview.
2. Studies investigating a combination of two interventions, where the non-pharmacological enhancer component of the intervention was not controlled for in the control condition (e.g., pharmacological enhancer + cognitive training vs. placebo).
3. Studies providing post-means only.
4. In case of multiple reports from the same study only one was included.
5. Although antipsychotic medication may also have a positive effect on cognition, this class of drugs was not included in this review as they are generally not taken to belong to the group of “cognitive enhancers”.

### Outcome measures

The current meta-analysis focused on cognitive outcomes specifically. First, we evaluated the effects of cognitive enhancers on overall cognition by including composite scores as provided by a cognitive test battery (for example Brief Assessment of Cognition in Schizophrenia (BACS)^36^ total score or MATRICS Consensus Cognitive Battery (MCCB)^37^ total score) or as calculated by the authors. Second, individual neuropsychological tests were grouped into seven cognitive domains, relevant for schizophrenia: 1. attention/vigilance, 2. processing speed (PS), 3. reasoning, 4. verbal learning and memory, 5. visual learning and memory, and 6. working memory (WM) [as recommended by MATRICS (Measurement and Treatment Research to Improve Cognition in Schizophrenia)], in addition to 7. verbal fluency (as measured by the majority of included studies). When a study applied multiple cognitive tests to assess the same cognitive domain, the primary outcome measure as defined by the authors was included in the meta-analysis. When the authors did not define the primary outcome, we selected the test most relevant to our defined cognitive domains. If studies reported multiple outcomes for a single cognitive test (for example, the Wisconsin Card Sorting Task (WCST) resulting in the number of completed categories, but also preservative errors), the outcome most commonly used across studies was used.

### Analyses and sub-analyses

In the overall analyses, whenever possible all identified study samples were included for each of the defined outcome measures (i.e., cognitive domains). Subsequently, study samples were grouped according to the neurotransmitter system of the cognitive enhancer studied (as described in section 2.1). Sub-analyses for pharmacological agents acting on the same or relevant pathway were conducted only when at least three different studies were identified, and by grouping the study samples for each specific/relevant pathway.

### Calculations

Effect sizes were computed using Comprehensive Meta-Analysis Version 2.0, Biostat.^38^ Hedges’s *g* was used to quantify effect sizes (ES) for the mean difference between change scores (end of treatment minus baseline) of the intervention group vs. placebo group. Although change scores are subject to increased error variance, we preferred these over pre- and post-treatment scores to avoid overestimation of the true effect size because of the pre- and -post-treatment correlation. When change scores were not provided by the authors, pre- and post-treatment scores were used. If not reported, pre- and post-treatment means and standard deviations (SDs), or exact *F*-, *t*- or *p-*values were used. Some studies had more than one follow-up time-point. Therefore, the last follow-up time-point of active treatment of the study sample was used. Single dose (i.e., challenge) studies were included only if they had pre- and post-assessments and analyses were run with and without these studies. Studies with multiple treatment groups (for example, different doses) and one placebo group were entered as individual study samples (*k*). As these treatment groups are dependent due to sharing a control group and the effective sample size is inflated,^38^ analyses that yielded significant results were repeated by splitting the shared placebo group into two or three groups with smaller sample size.

Studies were combined to calculate a mean weighted ES for each cognitive outcome measure, using a random effects model. Effect sizes were interpreted according to Cohen,^39^ with an ES of 0.2 indicating a small, 0.5 medium, and > 0.8 a large effect. To investigate whether studies could be combined to share a common population effect size, the *Q*-value and *I*^*2*^–statistic were evaluated for each analysis. The *Q*-statistic tests the existence of heterogeneity, and displays a chi-square distribution with *k*-1 degrees of freedom (*k* = number of studies), where *Q*-values higher than the degrees of freedom indicate significant between-studies variability. *I*^*2*^ reflects which proportion of the observed variance reflects differences in true effect sizes rather than sampling error, ranging from 0 to 100%. Values of 25%, 50%, and 75% can be interpreted as low, moderate, and high, respectively.^40^ Potential outlier studies were evaluated when heterogeneity exceeded 50%, which were defined as standardized residual *z*‐scores of effect sizes exceeding ± 1.96 (*p* < 0.05, two-tailed).

When interpreting meta-analytic outcomes, the possibility of an upward bias of the calculated effect sizes due to the omission of unpublished, nonsignificant studies must be taken into account.^41^ Potential publication bias was investigated by means of a visual inspection of the funnel plot and Egger’s test^42^ was evaluated when appropriate (i.e., analysis included a range of study sizes, with at least one of ‘‘medium’’ size (*p* < .05 two-tailed). Moreover, the fail-safe number of studies (*N*_*R*_) was calculated, providing an estimate of how many unpublished null findings would be needed to reduce an observed overall significant result to nonsignificance (the fail-safe number should be 5*k* + 10 or higher (*k* = number of studies in a meta-analysis) to rule out a file drawer problem.^41^

## Electronic supplementary material

Supplementary material

## Data Availability

The manuscript reports meta-analytic data based on individual original studies. The extracted data for the meta-analytic calculations are available upon request.

## Electronic supplementary material

**Supplementary information** accompanies the paper on the *npj Schizophrenia* website (10.1038/s41537-018-0064-6).
