# Supplementary material for: Efficacy of different types of cognitive enhancers for patients with schizophrenia: a meta-analysis
Source: NPJ Schizophr. 2018 Oct 25;4:22. doi: 10.1038/s41537-018-0064-6 (PMC6202388; doi:10.1038/s41537-018-0064-6)

Supplementary material S1
PRISMA diagram

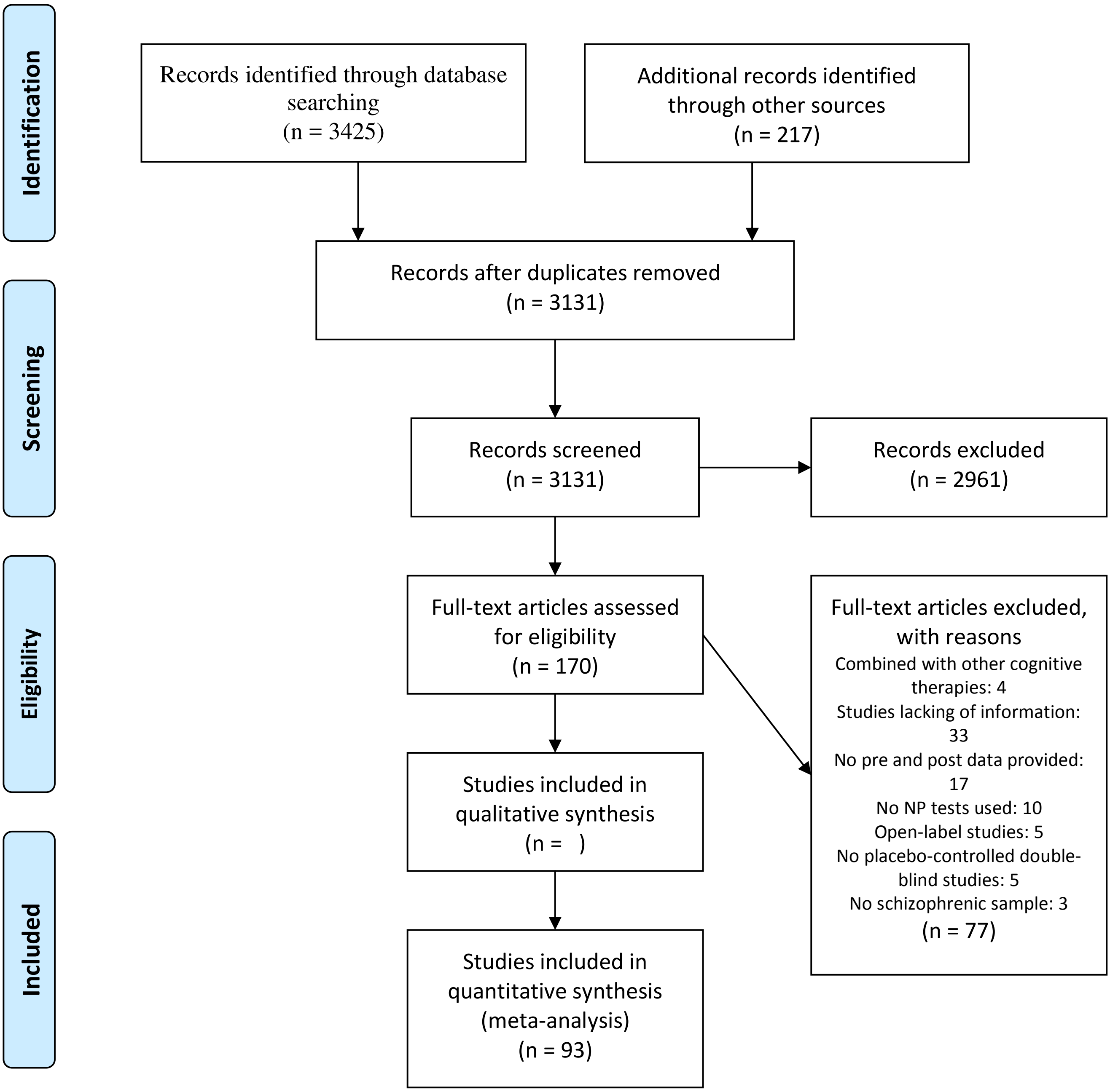

Supplementary material S2

Study	Cognitive outcome measures							
	**Overall cognition**	**Attention**	**Processing speed**	**Reasoning and problem solving**	**Verbal fluency**	**Verbal learning and memory**	**Visual learning and memory**	**Working memory**
Glutamatergic system								
Tsai 1998				X				
Goff 1999								
Tsai 1999				X				
Goff 2001		X	X	X	X	X	X	
Duncan 2004		X						X
Silver 2005	X	X					x	X
Buchanan 2007	X	X	X	X	X	X	x	X
Zoccali 2007				X				
Goff 2008a		X	X	X			X	X
Goff 2008b	X							
de Lucena 2009	X							
Lieberman 2009	X							
Marx 2009	X	X	X	X		X	X	X
Levkovitz 2010		X					X	X
Chengappa 2012				X		X		X
Lee 2012	X		X		X	X	X	
Vayisoglu 2012			X	X		X		X
Weiser 2012	X	X	X	X	X	X	X	X
D'Souza 2013		X	X	X		X		X
Lane 2013	X	X	X	X		X	X	X
Liu 2014		X	X	X		X	X	X
Schoemaker 2014	X	X	X	X		X	X	X
Kelly 2015	X	X		X		X	X	X
Lin 2015	X	X	X	X		X	X	X
Veerman 2016				X		X	X	X
Kantrowitz 2017	X	X	X	X		X	X	X
Mazinani 2017	X							

Cholinergic system

Friedman 2002		X				X		
Smith 2002			X				X	
Tugal 2004			X	X	X	X	X	X
Kumari 2006								X
Schubert 2006	X	X			X	X	X	X
Smith 2006		X						X
Fagerlund 2007				X		X		
Kohler 2007		X		X		X	X	
Lee B.J. 2007	X		X		X	X	X	X
Lee S.W. 2007	X		X		X	X	X	X
Akhondzadeh 2008				X		X	X	X
Barr 2008		X		X				X
Buchanan 2008	X	X	X			X	X	X
Dyer 2008		X		X				X
Freedman 2008		X	X	X		X	X	X
Keefe 2008	X	X	X	X	X	X	X	X
Shiina 2010		X		X			X	X
Hong 2011	X	X	X					
Lindenmayer 2011	X	X	X	X				X
Velligan 2012			X				X	X
Shim 2012		X		X				X
Zhang 2012	X				X	X	X	X
Deutsch 2013		X	X			X	X	X
Lieberman 2013	X	X	X	X		X	X	X
Umbricht 2014	X	X	X	X		X	X	X
Zhu 2014		X	X	X		X	X	X
Keefe 2015		X	X	X		X	X	X
Walling 2015	X							
Haig 2016a	X	X	X	X		X	X	X
Haig 2016b	X	X	X	X		X	X	X
Shoja Shafti & Azizi Khoei 2016	X							
Buchanan 2017	X	X	X	X		X	X	X

Serotoninergic system								
Sumiyoshi 2001				X		X		
Poyurovsky 2003		X	X	X			X	
Friedman 2005		X	X	X	X	X		X
Sumiyoshi 2007			X	X	X	X		X
Akhondzadeh 2009				X		X	X	
Berk 2009			X		X	X		X
Piskulic 2009			X	X		X		X
Stenberg 2010			X	X	X	X	X	x
Mico 2011				X	X			
Morozova 2012		X	X	X		X	X	X
Niitsu 2012		X		X			X	X
Morozova 2014		X	X					
Sheikhmoonesi 2015	X							
Samadi 2017				X				
Dopaminergic system								
Pietrzak 2010		X	X	X			X	
Kaphzan 2014	X							
Girgis 2016				X				
GABA-ergic system								
Lewis 2008		X			X	X	X	X
Buchanan 2011		X	X	X		X	X	X
Noradrenergic system								
Friedman 2001								X
Friedman 2008			X	X	X	X		X
Kelly 2009	X	X	X	X	X	X	X	X
Poyurovsky 2009		X	x	X			X	
Miscellaneous								

Sevy 2005		X			X	X	X	X
Pierre 2007		X	X			X		
Goff 2009	X	X			X	X		X
Kane 2010	X	X	X	X		X	X	X
Bobo 2011		X		X	X			X
Javitt 2012		X	X	X		X	X	X
Kane 2012		X	X	X		X		
Yi 2012			X	X	X	X		X
Lohr 2013	X		X					
Huerta-Ramos 2014		X	X		X			X
Lees 2017	X	X	X	X		X	x	X

Supplementary material S3

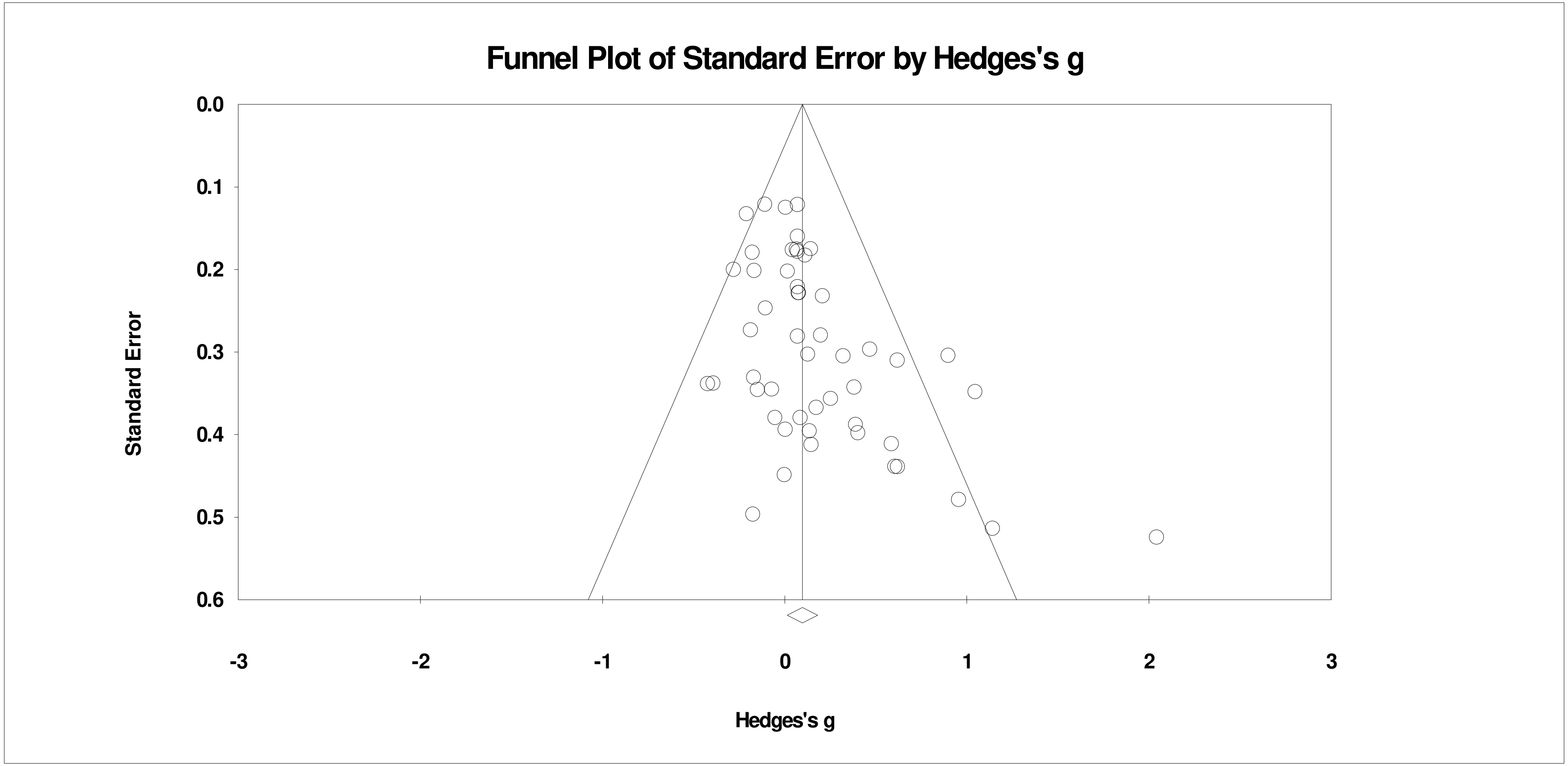

Supplementary material S4

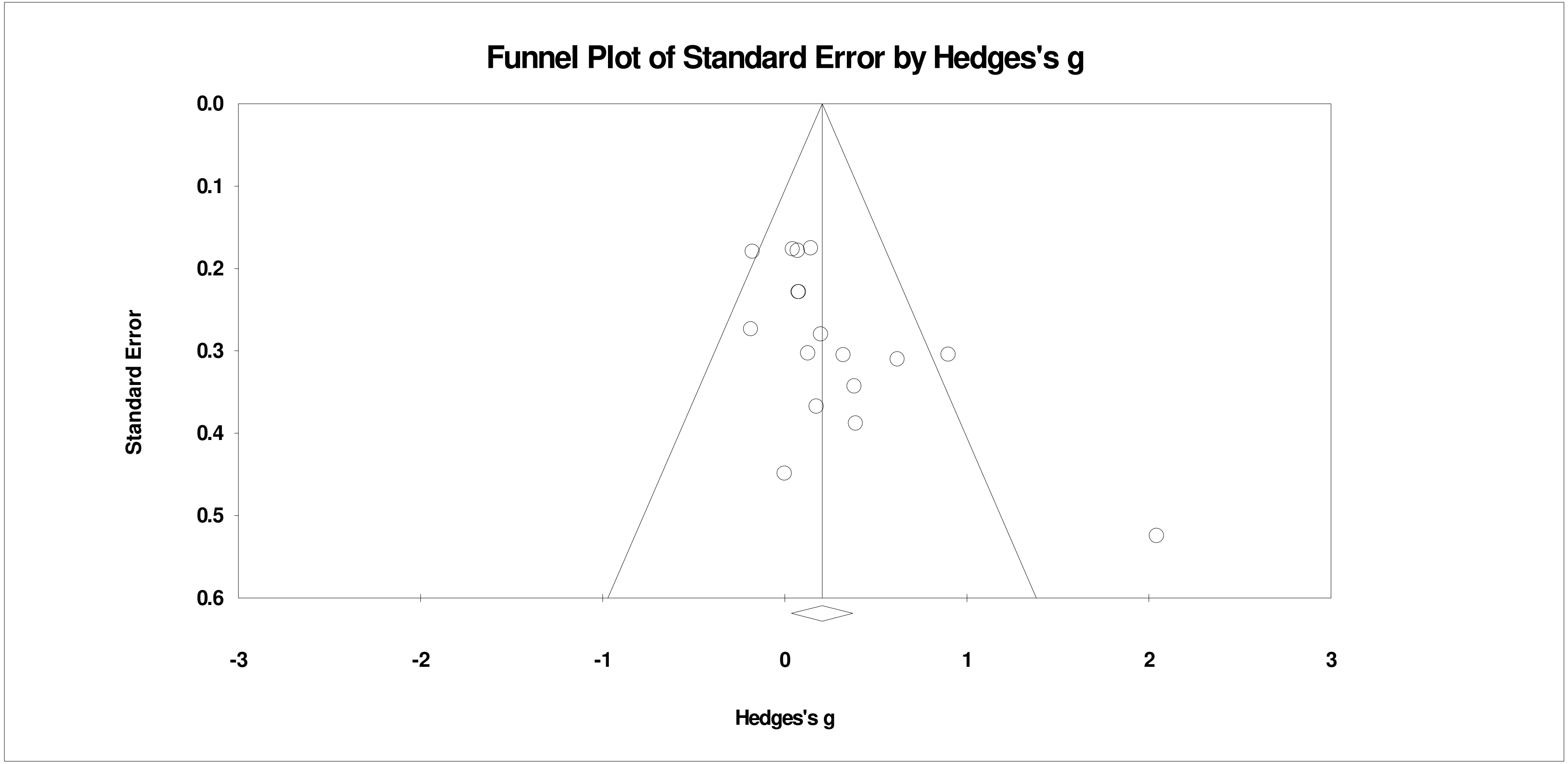

Supplementary material S5
Summary of the results of included study samples

Cognitive domain	Number of studies (*k*)	Patients (*N*)	ES Hedges's *g*	(95% CI)	*p*-value	*Q*-statistic (df)	I^2	Egger's test	Failsafe N_R
Glutamatergic drugs									
Overall cognition	17	1053	0.186	(0.040 to 0.340)	0.013	Q(16)=28.217, p=0.030	43.296	0.00836	36
Attention	18	1059	0.021	(-0.121 to 0.162)	0.775	Q(17)=13.365, p=0.711	0.000	0.79603	0
PS	17	1053	0.085	(-0.035 to 0.205)	0.163	Q(16)=7.390, p=0.965	0.000	0.25153	0
Reasoning	21	1189	0.090	(-0.036 to 0.217)	0.162	Q(20)=15.946, p=0.720	0.000	0.06339	0
Verbal fluency	5	326	-0.068	(-0.284 to 0.148)	0.537	Q(4)=1.562, p=0.816	0.000	0.35664	0
Verbal L&M	18	1073	0.085	(-0.042 to 0.212)	0.188	Q(17)=8.687, p=0.950	0.000	0.11478	0
Visual L&M	18	1072	0.074	(-0.048 to 0.196)	0.236	Q(17)=11.234, p=0.844	0.000	0.12292	0
WM	20	1162	0.133	(0.006 to 0.260)	0.040	Q(19)=20.049, p=0.392	5.233	0.14547	13
Subanalysis: Glycine site acting drugs									
Overall cognition	10	717	0.179	(-0.052 to 0.409)	0.128	Q(9)=3.840, p=0.922	0.000	0.03776	4
Attention	11	727	0.007	(-0.137 to 0.152)	0.919	Q(10)=8.327, p=0.597	0.000	0.59650	0
PS	10	735	0.109	(-0.035 to 0.252)	0.138	Q(9)=2.237, p=0.987	0.000	0.04268	0
Reasoning	12	767	0.087	(-0.054 to 0.227)	0.227	Q(11)=8.909, p=0.630	0.000	0.02908	0
Verbal fluency	3	282	-0.104	(-0.336 to 0.129)	0.382	Q(2)=0.565, p=0.754	0.000	0.11389	0
Verbal L&M	10	743	0.084	(-0.058 to 0.227)	0.248	Q(9)=3.623, p=0.934	0.000	0.55584	0
Visual L&M	9	694	0.061	(-0.087 to 0.208)	0.421	Q(8)=7.456, p=0.488	0.000	0.35601	0
WM	11	737	0.033	(-0.110 to 0.177)	0.648	Q(10)=4.547, p=0.919	0.000	0.56687	0
Subanalysis: AMPA site									
Overall cognition	1	50	0.200	(-0.565 to 0.964)	0.609	Q(0)=0.000, p=1.000	0.000	n/a	n/a
Attention	5	262	0.144	(-0.097 to 0.385)	0.242	Q(4)=2.198, p=0.699	0.000	0.37836	0
PS	4	241	0.095	(-0.157 to 0.346)	0.461	Q(3)=2.891, p=0.409	0.000	0.06015	0

Reasoning	4	241	0.037	(-0.214 to 0.288)	0.773	Q(3)=2.806, p=0.423	0.000	0.82539	0
Verbal fluency	1	18	0.375	(-0.567 to 1.316)	0.435	Q(0)=0.000, p=1.000	0.000	n/a	n/a
Verbal L&M	3	147	0.045	(-0.278 to 0.368)	0.785	Q(2)=3.806, p=0.149	47.447	0.40400	0
Visual L&M	5	262	0.110	(-0.131 to 0.351)	0.373	Q(4)=3.073, p=0.546	0.000	0.02803	0
WM	4	244	0.279	(0.026 to 0.532)	0.030	Q(3)=8.911, p=0.030	66.334	0.00382	5
Subanalysis: Memantine/Amantadine									
Overall cognition	5	268	0.342	(-0.019 to 0.703)	0.063	Q(4)=24.186, p=0.000	83.461	0.08067	6
Attention	1	52	-0.260	(-0.798 to 0.277)	0.343	Q(0)=0.000, p=1.000	0.000	n/a	n/a
PS	1	26	0.133	(-0.621 to 0.888)	0.729	Q(0)=0.000, p=1.000	0.000	n/a	n/a
Reasoning	1	49	-0.222	(-0.775 to 0.331)	0.432	Q(0)=0.000, p=1.000	0.000	n/a	n/a
Verbal fluency	1	26	0.024	(-0.730 to 0.777)	0.950	Q(0)=0.000, p=1.000	0.000	n/a	n/a
Verbal L&M	2	75	0.165	(-0.281 to 0.611)	0.469	Q(1)=0.198, p=0.657	0.000	n/a	n/a
Visual L&M	3	98	0.113	(-0.275 to 0.501)	0.567	Q(2)=0.102, p=0.950	0.000	0.27666	0
WM	2	72	0.306	(-0.150 to 0.762)	0.189	Q(1)=0.598, p=0.439	0.000	n/a	n/a
All cholinergic agents (smokers and non-smokers)									
Overall cognition	21	2136	0.077	(-0.042 to 0.197)	0.204	Q(20)=32.852, p=0.035	39.120	0.00036	11
Attention	27	2151	0.009	(-0.097 to 0.115)	0.868	Q(26)=51.952, p=0.002	49.954	0.44374	0
PS	27	2508	0.001	(-0.077 to 0.079)	0.984	Q(26)=28.869, p=0.317	9.939	0.03173	0
Reasoning	23	1909	-0.014	(-0.120 to 0.093)	0.798	Q(22)=33.321, p=0.057	33.976	0.21284	0
Verbal fluency	8	357	-0.138	(-0.344 to 0.067)	0.187	Q(7)=10.391, p=0.167	32.632	0.10853	0
Verbal L&M	28	1910	0.073	(-0.026 to 0.171)	0.147	Q(27)=45.805, p=0.013	41.055	0.21900	0
Visual L&M	30	2429	0.034	(-0.048 to 0.116)	0.417	Q(29)=33.498, p=0.258	13.427	0.32901	0
WM	34	2612	0.041	(-0.048 to 0.130)	0.364	Q(33)=41.868, p=0.138	21.182	0.03870	0

Subanalysis: excluded challenge studies

Overall cognition	21	2136	0.075	(-0.043 to 0.193)	0.211	Q(20)=32.852, p=0.035	39.120	0.00036	11
Attention	25	2045	-0.011	(-0.142 to 0.119)	0.864	Q(24)=47.940, p=0.003	49.937	0.61001	0
PS	25	2238	-0.009	(-0.092 to 0.068)	0.835	Q(24)=25.723, p=0.367	6.700	0.05778	0
Reasoning	22	1853	-0.041	(-0.164 to 0.083)	0.520	Q(21)=29.536, p=0.102	28.901	0.12209	0
Verbal fluency	8	357	-0.138	(-0.344 to 0.067)	0.187	Q(7)=10.391, p=0.167	32.632	0.10853	0
Verbal L&M	28	1910	0.072	(-0.026 to 0.169)	0.148	Q(27)=45.805, p=0.013	41.055	0.21900	0
Visual L&M	29	2349	0.034	(-0.056 to 0.125)	0.458	Q(28)=32.964, p=0.237	15.058	0.36351	0
WM	32	2257	0.026	(-0.056 to 0.108)	0.534	Q(31)=36.945, p=0.213	16.091	0.04593	0

Subanalysis: nicotinic agents (alpha7 nicotinic agents and alpha4 nicotinic agents)

Overall cognition	13	1676	0.013	(-0.088 to 0.113)	0.803	Q(12)=12.508, p=0.406	4.065	0.04259	0
Attention	15	1523	0.032	(-0.097 to 0.161)	0.629	Q(14)=21.887, p=0.081	36.034	0.81995	0
PS	17	1919	-0.051	(-0.149 to 0.047)	0.308	Q(16)=18.650, p=0.287	14.208	0.07615	0
Reasoning	13	1418	-0.009	(-0.113 to 0.094)	0.863	Q(12)=10.874, p=0.540	0.000	0.11888	0
Verbal fluency	3	58	0.054	(-0.553 to 0.662)	0.861	Q(2)=1.057, p=0.589	0.000	0.97213	0
Verbal L&M	15	1339	0.058	(-0.103 to 0.219)	0.482	Q(14)=25.211, p=0.033	44.468	0.25065	0
Visual L&M	19	1857	0.012	(-0.079 to 0.102)	0.802	Q(18)=16.704, p=0.544	0.000	0.10634	0
WM	20	1947	-0.012	(-0.101 to 0.076)	0.784	Q(19)=10.813, p=0.930	0.000	0.71375	0

Subanalysis: alpha7 nicotinic agents

Overall cognition	12	1612	0.040	(-0.112 to 0.193)	0.603	Q(11)=12.280, p=0.343	10.427	0.02888	0
Attention	13	1368	0.035	(-0.128 to 0.198)	0.673	Q(12)=21.795, p=0.040	44.941	0.87370	0
PS	12	1279	0.005	(-0.105 to 0.115)	0.935	Q(11)=10.401, p=0.495	0.000	0.35107	0
Reasoning	12	1327	-0.020	(-0.169 to 0.129)	0.791	Q(11)=10.607, p=0.477	0.000	0.14995	0
Verbal fluency	3	58	0.054	(-0.441 to 0.550)	0.831	Q(2)=1.057, p=0.589	0.000	0.97213	0

Verbal L&M	15	1339	0.058	(-0.103 to 0.219)	0.482	Q(14)=25.211, p=0.033	44.468	0.25065	0
Visual L&M	16	1372	0.061	(-0.057 to 0.180)	0.312	Q(15)=13.942, p=0.530	0.000	0.24431	0
WM	16	1372	0.042	(-0.079 to 0.153)	0.498	Q(15)=6.793, p=0.963	0.000	0.78993	0
Subanalysis: alpha4 nicotinic agents									
Overall cognition	1	64	-0.104	(-0.703 to 0.495)	0.734	Q(0)=0.000, p=1.000	0.000	n/a	n/a
Attention	2	155	-0.004	(-0.439 to 0.430)	0.984	Q(1)=0.034, p=0.854	0.000	n/a	n/a
PS	5	640	-0.175	(-0.331 to -0.020)	0.027	Q(4)=4.802, p=0.308	16.699	0.10042	0
Reasoning	1	91	-0.113	(-0.643 to 0.417)	0.676	Q(0)=0.000, p=1.000	0.000	n/a	n/a
Verbal fluency	0	n/a	n/a	n/a	n/a	n/a	n/a	n/a	n/a
Verbal L&M	0	n/a	n/a	n/a	n/a	n/a	n/a	n/a	n/a
Visual L&M	3	485	-0.104	(-0.312 to 0.104)	0.328	Q(2)=0.569, p=0.753	0.000	0.87766	0
WM	4	575	-0.147	(-0.338 to 0.043)	0.128	Q(3)=0.371, p=0.946	0.000	0.51217	0
Subanalysis: AchIE									
Overall cognition	3	285	0.177	(-0.173 to 0.526)	0.321	Q(2)=13.525, p=0.001	85.213	0.23976	0
Attention	5	354	0.005	(-0.315 to 0.324)	0.975	Q(4)=6.073, p=0.194	34.131	0.06466	0
PS	4	314	0.103	(-0.116 to 0.322)	0.358	Q(3)=0.783, p=0.854	0.000	0.75609	0
Reasoning	6	353	-0.111	(-0.395 to 0.174)	0.447	Q(5)=5.999, p=0.306	16.653	0.43079	0
Verbal fluency	3	261	-0.237	(-0.480 to 0.006)	0.056	Q(2)=6.294, p=0.043	68.225	0.70091	0
Verbal L&M	9	430	0.116	(-0.153 to 0.385)	0.400	Q(8)=18.263, p=0.019	56.195	0.86053	0
Visual L&M	6	369	0.148	(-0.085 to 0.381)	0.212	Q(5)=14.281, p=0.014	64.988	0.97237	0
WM	6	364	0.260	(0.024 to 0.496)	0.031	Q(5)=14.479, p=0.013	65.468	0.10486	9

Subanalysis: Galantamine

Overall cognition	5	175	0.288	(-0.053 to 0.629)	0.097	Q(4)=4.035, p=0.401	0.859	0.26102	1
Attention	5	168	-0.272	(-0.642 to 0.099)	0.141	Q(4)=14.003, p=0.007	71.434	0.14853	0
PS	4	159	0.272	(-0.035 to 0.579)	0.082	Q(3)=1.031, p=0.794	0.000	0.16613	0
Reasoning	3	82	0.005	(-0.476 to 0.487)	0.983	Q(2)=12.267, p=0.002	83.696	0.09829	0
Verbal fluency	2	38	0.197	(-0.417 to 0.811)	0.529	Q(1)=0.678, p=0.410	0.000	n/a	n/a
Verbal L&M	4	141	0.297	(-0.104 to 0.699)	0.147	Q(3)=0.175, p=0.982	0.000	0.64360	0
Visual L&M	4	141	-0.060	(-0.400 to 0.281)	0.732	Q(3)=0.515, p=0.915	0.000	0.86833	0
WM	6	193	0.044	(-0.240 to 0.335)	0.775	Q(5)=8.228, p=0.144	39.235	0.73593	0

All serotonergic agents

Overall cognition	1	49	0.073	(-0.561 to 0.706)	0.822	Q(0)=0.000, p=1.000	0.000	n/a	n/a
Attention	5	192	0.229	(-0.080 to 0.537)	0.146	Q(4)=2.296, p=0.682	0.000	0.71602	0
PS	8	289	0.014	(-0.214 to 0.241)	0.907	Q(7)=3.820, p=0.800	0.000	0.65256	0
Reasoning	11	398	0.037	(-0.169 to 0.243)	0.726	Q(10)=5.424, p=0.861	0.000	0.40377	0
Verbal fluency	5	208	-0.019	(-0.287 to 0.248)	0.888	Q(4)=2.490, p=0.646	0.000	0.81200	0
Verbal L&M	8	283	0.003	(-0.235 to 0.241)	0.980	Q(7)=8.496, p=0.291	17.613	0.26701	0
Visual L&M	5	179	0.124	(-0.171 to 0.419)	0.409	Q(4)=11.042, p=0.062	63.774	0.01824	0
WM	8	304	-0.027	(-0.270 to 0.215)	0.824	Q(7)=8.323, p=0.305	15.899	0.42442	0

Subanalysis: only (partial) 5-HT1A agonist

Overall cognition	1	49	0.073	(-0.479 to 0.624)	0.797	Q(0)=0.000, p=1.000	0.000	n/a	n/a
Attention	0	n/a	n/a	n/a	n/a	n/a	n/a	n/a	n/a

PS	2	77	-0.031	(-0.469 to 0.407)	0.890	Q(1)=0.556, p=0.456	0.000	n/a	n/a
Reasoning	3	103	0.074	(-0.307 to 0.454)	0.704	Q(2)=2.106, p=0.349	5.039	0.68349	0
Verbal fluency	1	59	-0.068	(-0.572 to 0.436)	0.791	Q(0)=0.000, p=1.000	0.000	n/a	n/a
Verbal L&M	3	103	0.040	(-0.463 to 0.542)	0.877	Q(2)=2.989, p=0.224	33.092	0.53982	0
Visual L&M	0	n/a	n/a	n/a	n/a	n/a	n/a	n/a	n/a
WM	2	77	-0.054	(-0.570 to 0.461)	0.836	Q(1)=1.677, p=0.195	40.353	n/a	n/a

Subanalysis: only antidepressants

Overall cognition	0	n/a	n/a	n/a	n/a	n/a	n/a	n/a	n/a
Attention	3	109	0.172	(-0.196 to 0.541)	0.359	Q(2)=0.096, p=0.953	0.000	0.20861	0
PS	4	129	0.060	(-0.279 to 0.400)	0.727	Q(3)=2.758, p=0.430	0.000	0.83405	0
Reasoning	5	186	0.002	(-0.280 to 0.285)	0.986	Q(4)=2.648, p=0.618	0.000	0.81320	0
Verbal fluency	4	149	0.000	(-0.316 to 0.316)	1.000	Q(3)=2.440, p=0.486	0.000	0.01175	0
Verbal L&M	3	109	0.071	(-0.410 to 0.549)	0.777	Q(2)=2.587, p=0.274	22.686	0.06473	0
Visual L&M	3	108	0.092	(-0.598 to 0.783)	0.792	Q(2)=8.406, p=0.015	76.206	0.01893	0
WM	4	156	0.097	(-0.258 to 0.451)	0.593	Q(3)=2.649, p=0.449	0.000	0.85270	0

All dopaminergic agents

Overall cognition	1	45	0.469	(-0.192 to 1.129)	0.164	Q(0)=0.000, p=1.000	0.000	n/a	n/a
Attention	1	64	0.464	(-0.103 to 1.031)	0.109	Q(0)=0.000, p=1.000	0.000	n/a	n/a
PS	1	64	0.188	(-0.297 to 0.673)	0.448	Q(0)=0.000, p=1.000	0.000	n/a	n/a
Reasoning	3	130	0.337	(-0.030 to 0.705)	0.072	Q(2)=0.165, p=0.921	0.000	0.22277	0
Verbal fluency	0	n/a	n/a	n/a	n/a	n/a	n/a	n/a	n/a
Verbal L&M	0	n/a	n/a	n/a	n/a	n/a	n/a	n/a	n/a
Visual L&M	1	64	0.292	(-0.204 to 0.789)	0.248	Q(0)=0.000, p=1.000	0.000	n/a	n/a

WM	0	n/a	n/a	n/a	n/a	n/a	n/a	n/a	n/a
All GABA-ergic agents									
Overall cognition	3	82	-0.161	(-0.623 to 0.301)	0.495	Q(2)=0,003, p=0.999	0.000	0.73160	0
Attention	3	82	0.281	(-0.180 to 0.742)	0.232	Q(2)=2.641, p=0.267	24.276	0.03926	0
PS	2	67	-0.048	(-0.516 to 0.420)	0.839	Q(1)=0,001, p=0.972	0.000	n/a	n/a
Reasoning	2	67	-0.346	(-0.845 to 0.154)	0.175	Q(1)=0.043, p=0.835	0.000	n/a	n/a
Verbal fluency	1	15	-0.240	(-1.216 to 0.736)	0.630	Q(0)=0.000, p=1.000	0.000	n/a	n/a
Verbal L&M	3	82	-0.248	(-0.684 to 0.188)	0.264	Q(2)=0.462, p=0.794	0.000	0.35751	0
Visual L&M	3	82	-0.381	(-0.811 to 0.049)	0.082	Q(2)=0.042, p=0.979	0.000	0.39318	0
WM	3	82	0.284	(-0.162 to 0.730)	0.212	Q(2)=1.531, p=0.465	0.000	0.28046	0
All noradrenergic agents									
Overall cognition	1	22	0.148	(-0.719 to 1.014)	0.739	Q(0)=0.000, p=1.000	0.000	n/a	n/a
Attention	2	55	-0.151	(-0.708 to 0.406)	0.595	Q(1)=1.676, p=0.195	40.337	n/a	n/a
PS	3	70	0.055	(-0.399 to 0.509)	0.813	Q(2)=0.877, p=0.645	0.000	0.31157	0
Reasoning	3	70	0.084	(-0.392 to 0.559)	0.730	Q(2)=1.150, p=0.563	0.000	0.23840	0
Verbal fluency	2	37	0.248	(-0.371 to 0.868)	0.432	Q(1)=0.011, p=0.915	0.000	n/a	n/a
Verbal L&M	2	37	0.313	(-0.320 to 0.945)	0.333	Q(1)=0.085, p=0.771	0.000	n/a	n/a
Visual L&M	2	55	-0.061	(-0.580 to 0.458)	0.818	Q(1)=0.006, p=0.936	0.000	n/a	n/a
WM	3	75	0.307	(-0.157 to 0.772)	0.194	Q(2)=2.872, p=0.238	0.000	0.27502	0
All Miscellaneous agents									
Overall cognition	7	248	-0.076	(-0.351 to 0.200)	0.590	Q(6)=0.918, p=0.989	0.000	0.68097	0
Attention	15	832	-0.144	(-0.305 to 0.017)	0.080	Q(14)=8.880, p=0.839	0.000	0.61247	0
PS	13	731	-0.062	(-0.205 to 0.081)	0.395	Q(12)=3.601, p=0.990	0.000	0.18206	0

Reasoning	11	729	-0.088	(-0.256 to 0.079)	0.300	Q(10)=23.717, p=0.008	57.836	0.74505	0
Verbal fluency	6	191	0.068	(-0.210 to 0.347)	0.632	Q(5)=5.044, p=0.327	13.605	0.38257	0
Verbal L&M	15	805	-0.101	(-0.248 to 0.047)	0.180	Q(14)=11.656, p=0.641	0.000	0.30070	0
Visual L&M	7	252	0.080	(-0.166 to 0.326)	0.525	Q(6)=5.558, p=0.474	0.000	0.35392	0
WM	12	414	-0.059	(-0.261 to 0.143)	0.565	Q(11)=12.915, p=0.299	14.931	0.63934	0
Subanalysis: excluded challenge studies									
Overall cognition	4	100	0.043	(-0.336 to 0.423)	0.823	Q(3)=0.141, p=0.986	0.000	0.57824	0
Attention	12	684	-0.157	(-0.306 to -0.009)	0.038	Q(11)=10.718, p=0.467	0.000	0.37489	0
PS	12	651	-0.073	(-0.224 to 0.079)	0.348	Q(11)=6.066, p=0.869	0.000	0.18106	0
Reasoning	10	649	-0.122	(-0.400 to 0.156)	0.389	Q(9)=24.862, p=0.003	63.801	0.76757	0
Verbal fluency	4	123	0.250	(-0.097 to 0.598)	0.158	Q(3)=2.752, p=0.432	0.000	0.28443	0
Verbal L&M	12	657	-0.124	(-0.275 to 0.028)	0.110	Q(11)=10.798, p=0.460	0.000	0.28066	0
Visual L&M	6	127	0.205	(-0.088 to 0.498)	0.170	Q(5)=3.222, p=0.666	0.000	0.25814	0
WM	9	266	-0.032	(-0.276 to 0.211)	0.795	Q(8)=8.458, p=0.390	5.412	0.06932	0
Subanalysis: All (ar)modafinil agents									
Overall cognition	5	180	0.054	(-0.269 to 0.377)	0.741	Q(4)=0.151, p=0.997	0.000	0.71539	0
Attention	10	677	-0.134	(-0.284 to 0.071)	0.081	Q(9)=12.051, p=0.210	25.318	0.54984	0
PS	9	623	-0.068	(-0.223 to 0.088)	0.393	Q(8)=5.573, p=0.695	0.000	0.12588	0
Reasoning	8	637	-0.135	(-0.450 to 0.178)	0.395	Q(7)=24.813, p=0.001	71.788	0.64389	0
Verbal fluency	2	78	0.423	(-0.017 to 0.864)	0.059	Q(1)=0.604, p=0.437	0.000	n/a	n/a
Verbal L&M	9	619	-0.050	(-0.207 to 0.106)	0.530	Q(8)=7.744, p=0.459	0.000	0.01529	0
Visual L&M	5	179	-0.001	(-0.289 to 0.287)	0.993	Q(4)=4.008, p=0.405	0.201	0.44692	0
WM	6	237	-0.038	(-0.337 to 0.260)	0.801	Q(5)=8.013, p=0.156	37.600	0.72499	0

n/a –not applicable; at least 3 study samples should be included to run publication bias procedures.

Supplementary material S6

Attention

Group by System	Study name	Agent	Hedges's g	Lower limit	Upper limit	p-Value	Std Residual
a GLUTAMATERGIC SYSTEM	Goff 2001	Ampakine CX516	0.605	-0.349	1.559	0.214	1.16
a GLUTAMATERGIC SYSTEM	Duncan 2004	D-Cycloserine	-0.999	-2.018	0.021	0.055	-1.90
a GLUTAMATERGIC SYSTEM	Silver 2005	Amantadine	-0.260	-0.798	0.277	0.343	-0.93
a GLUTAMATERGIC SYSTEM	Buchanan 2007 g	Glycine	-0.107	-0.580	0.367	0.659	-0.47
a GLUTAMATERGIC SYSTEM	Buchanan 2007	D-Cycloserine	-0.216	-0.676	0.244	0.357	-0.89
a GLUTAMATERGIC SYSTEM	Goff 2008	Ampakine CX516	0.000	-0.401	0.401	1.000	-0.09
a GLUTAMATERGIC SYSTEM	Marx 2009	Pregnenolone	-0.373	-1.262	0.515	0.410	-0.84
a GLUTAMATERGIC SYSTEM	Levkowitz 2010	Minocycline	0.192	-0.655	1.039	0.657	0.38
a GLUTAMATERGIC SYSTEM	Weiser 2012	D-serine	-0.043	-0.422	0.335	0.823	-0.28
a GLUTAMATERGIC SYSTEM	D'Souza 2013	D-serine	0.127	-0.404	0.658	0.639	0.36
a GLUTAMATERGIC SYSTEM	Lane 2013	Benzoate	0.246	-0.308	0.800	0.384	0.73
a GLUTAMATERGIC SYSTEM	Liu 2014	Minocycline	0.303	-0.137	0.742	0.177	1.10
a GLUTAMATERGIC SYSTEM	Schoemaker 2014 12-16mg	Org 25935	0.136	-0.208	0.479	0.439	0.53
a GLUTAMATERGIC SYSTEM	Schoemaker 2014 4-8mg	Org 25935	-0.073	-0.423	0.277	0.685	-0.43
a GLUTAMATERGIC SYSTEM	Kelly 2015	Minocycline	-0.007	-0.555	0.540	0.979	-0.09
a GLUTAMATERGIC SYSTEM	Lin 2015 s	Sarcosine	-0.050	-0.643	0.544	0.870	-0.21
a GLUTAMATERGIC SYSTEM	Lin 2015	Sarcosine + Benzoate	0.360	-0.238	0.959	0.238	1.03
a GLUTAMATERGIC SYSTEM	Kantrowitz 2017	D-serine	0.232	-0.490	0.953	0.529	0.54
a GLUTAMATERGIC SYSTEM			0.021	-0.121	0.162	0.775	
b CHOLINERGIC SYSTEM	Friedman 2002 10mg	Donepezil	0.764	-0.069	1.596	0.072	1.69
b CHOLINERGIC SYSTEM	Friedman 2002 5mg	Donepezil	0.183	-0.569	0.935	0.633	0.43
b CHOLINERGIC SYSTEM	Schubert 2006	Galantamine	0.452	-0.488	1.392	0.346	0.89
b CHOLINERGIC SYSTEM	Smith 2006	Nicotine	0.386	-0.165	0.937	0.169	1.21
b CHOLINERGIC SYSTEM	Kohler 2007	Donepezil	0.149	-0.657	0.954	0.718	0.32
b CHOLINERGIC SYSTEM	Barr 2008	Nicotine	0.370	-0.151	0.891	0.164	1.21
b CHOLINERGIC SYSTEM	Buchanan 2008	Galantamine	-0.989	-1.481	-0.497	0.000	-3.51
b CHOLINERGIC SYSTEM	Dyer 2008	Galantamine	-0.510	-1.364	0.345	0.242	-1.14
b CHOLINERGIC SYSTEM	Keefe 2008	Donepezil	-0.233	-0.494	0.028	0.080	-1.28
b CHOLINERGIC SYSTEM	Freedman 2008 300mg	DMXB-A	-0.028	-0.520	0.463	0.910	-0.13
b CHOLINERGIC SYSTEM	Freedman 2008 150mg	DMXB-A	0.260	-0.234	0.754	0.302	0.88
b CHOLINERGIC SYSTEM	Shiina 2010	Tropisetron	-0.238	-0.907	0.430	0.485	-0.67
b CHOLINERGIC SYSTEM	Hong 2011	Varenicline	-0.037	-0.521	0.447	0.882	-0.16
b CHOLINERGIC SYSTEM	Lindenmayer 2011	Galantamine	-0.069	-0.746	0.608	0.842	-0.21
b CHOLINERGIC SYSTEM	Shim 2012	Varenicline	0.023	-0.385	0.430	0.914	0.05
b CHOLINERGIC SYSTEM	Lieberman 2013	TC-5619	0.247	-0.069	0.562	0.125	1.13
b CHOLINERGIC SYSTEM	Deutsch 2013	nAChR agonist	0.368	-0.228	0.964	0.226	1.08
b CHOLINERGIC SYSTEM	Umbricht 2014 50mg	RG3487	-0.579	-0.983	-0.176	0.005	-2.39
b CHOLINERGIC SYSTEM	Umbricht 2014 15mg	RG3487	-0.309	-0.702	0.084	0.123	-1.32
b CHOLINERGIC SYSTEM	Umbricht 2014 5mg	RG3487	-0.060	-0.457	0.337	0.768	-0.28
b CHOLINERGIC SYSTEM	Zhu 2014	Donepezil	-0.146	-0.682	0.391	0.595	-0.51
b CHOLINERGIC SYSTEM	Keefe 2015 0.9mg	Encenicline	0.410	0.036	0.783	0.032	1.72
b CHOLINERGIC SYSTEM	Keefe 2015 0.27mg	Encenicline	0.023	-0.345	0.391	0.904	0.06
b CHOLINERGIC SYSTEM	Haig 2016 25mg	ABT-126	0.180	-0.179	0.540	0.325	0.75
b CHOLINERGIC SYSTEM	Haig 2016 10mg	ABT-126	0.165	-0.180	0.510	0.348	0.71
b CHOLINERGIC SYSTEM	Haig 2016b	ABT-126	-0.027	-0.273	0.220	0.832	-0.19
b CHOLINERGIC SYSTEM	Buchanan 2017	Galantamine	0.360	-0.343	1.062	0.315	0.92
b CHOLINERGIC SYSTEM			0.009	-0.097	0.115	0.868	
c SEROTONINERGIC SYSTEM	Poyurovsky 2003	Mianserine	0.281	-0.498	1.060	0.480	0.13
c SEROTONINERGIC SYSTEM	Friedman 2005	Citalopram	0.141	-0.482	0.764	0.658	-0.28
c SEROTONINERGIC SYSTEM	Morozova 2012	Latrepirdine	0.000	-0.601	0.601	1.000	-0.76
c SEROTONINERGIC SYSTEM	Niitsu 2012 50mg	Fluvoxamine	0.141	-0.422	0.704	0.623	-0.31
c SEROTONINERGIC SYSTEM	Morozova 2014	AVN-211	0.622	0.003	1.241	0.049	1.27
c SEROTONINERGIC SYSTEM			0.229	-0.080	0.537	0.146	
e DOPAMINERGIC SYSTEM	Pietrzak 2010	D-amphetamine	0.464	-0.027	0.955	0.064	0.00
e DOPAMINERGIC SYSTEM			0.464	-0.103	1.031	0.109	
f GABA-ERGIC SYSTEM	Lewis 2008	MK-0777	1.046	0.004	2.088	0.049	1.53
f GABA-ERGIC SYSTEM	Buchanan 2011 16mg	MK-0777	0.052	-0.596	0.700	0.875	-0.84
f GABA-ERGIC SYSTEM	Buchanan 2011 6mg	MK-0777	0.175	-0.503	0.853	0.613	-0.36
f GABA-ERGIC SYSTEM			0.281	-0.180	0.742	0.232	
g NORADRENERGIC SYSTEM	Kelly 2009	Atomoxetine	0.252	-0.558	1.063	0.542	1.21
g NORADRENERGIC SYSTEM	Poyurovsky 2009	Norepinephrine	-0.444	-1.119	0.230	0.197	-1.21
g NORADRENERGIC SYSTEM			-0.151	-0.708	0.406	0.595	
h MISCELLANEOUS	Sevy 2005	Modafinil	0.302	-0.543	1.147	0.483	1.00
h MISCELLANEOUS	Pierre 2007	Modafinil	0.144	-0.697	0.984	0.738	0.65
h MISCELLANEOUS	Goff 2009 100mg	Sildenafil	-0.373	-1.035	0.290	0.270	-0.64
h MISCELLANEOUS	Goff 2009 50mg	Sildenafil	-0.381	-1.044	0.281	0.259	-0.66
h MISCELLANEOUS	Kane 2010 100mg	Armodafinil	0.117	-0.616	0.849	0.755	0.66
h MISCELLANEOUS	Kane 2010 150mg	Armodafinil	-0.177	-0.937	0.583	0.648	-0.08
h MISCELLANEOUS	Kane 2010 50mg	Armodafinil	-0.277	-1.013	0.459	0.460	-0.34
h MISCELLANEOUS	Bobo 2011	Armodafinil	-0.493	-1.009	0.022	0.061	-1.21
h MISCELLANEOUS	Javitt 2012 30mg	Davunetide	-0.136	-0.768	0.495	0.673	0.02
h MISCELLANEOUS	Javitt 2012 5mg	Davunetide	-0.074	-0.714	0.566	0.821	0.20
h MISCELLANEOUS	Kane 2012 250mg	Armodafinil	-0.069	-0.398	0.259	0.678	0.36
h MISCELLANEOUS	Kane 2012 150mg	Armodafinil	0.053	-0.277	0.382	0.755	0.95
h MISCELLANEOUS	Kane 2012 200mg	Armodafinil	-0.530	-0.867	-0.194	0.002	-1.85
h MISCELLANEOUS	Huerta-Ramos 2014	Raloxifene	0.063	-0.918	1.044	0.900	0.40
h MISCELLANEOUS	Lees 2017	Modafinil	0.131	-0.304	0.565	0.555	1.09
h MISCELLANEOUS			-0.144	-0.305	0.017	0.080	
Overall			0.005	-0.066	0.076	0.893	

Hedges's g and 95% CI

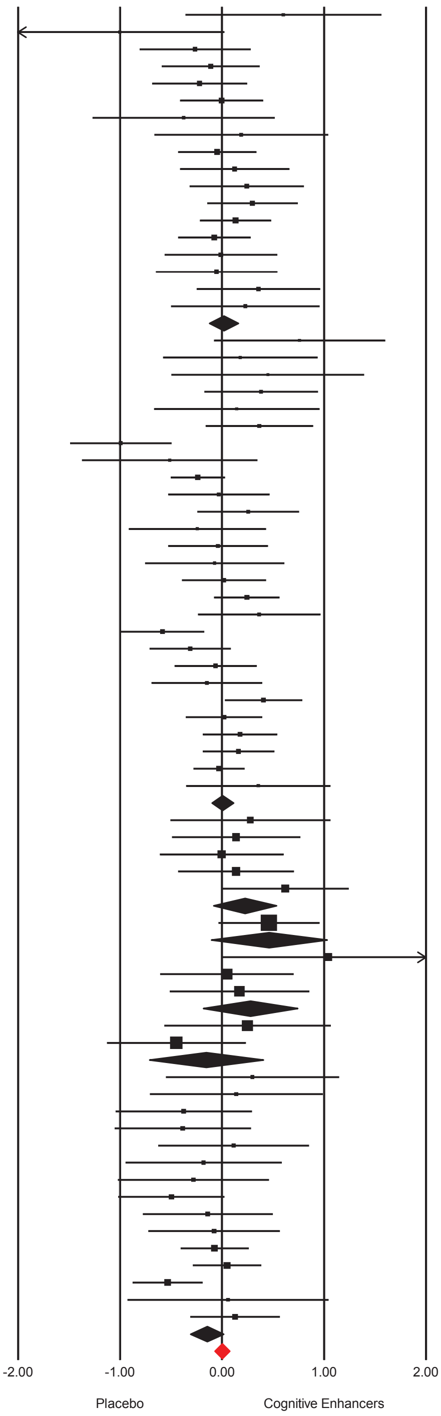

Processing speed

Group by System	Study name	Agent	Hedges's g	Lower limit	Upper limit	p-Value	Std Residual
a GLUTAMATERGIC SYSTEM	Goff 2001	Ampakine CX516	0.748	-0.217	1.712	0.129	1.36
a GLUTAMATERGIC SYSTEM	Goff 2008	Ampakine CX516	-0.104	-0.505	0.298	0.613	-0.97
a GLUTAMATERGIC SYSTEM	Buchanan 2007 g	Glycine	0.177	-0.267	0.620	0.434	0.42
a GLUTAMATERGIC SYSTEM	Buchanan 2007	D-Cycloserine	0.171	-0.264	0.606	0.441	0.40
a GLUTAMATERGIC SYSTEM	Marx 2009	Pregnenolone	-0.212	-1.095	0.671	0.638	-0.67
a GLUTAMATERGIC SYSTEM	Lee 2012	Memantine	0.133	-0.621	0.888	0.729	0.13
a GLUTAMATERGIC SYSTEM	Vayisoglu 2012	Lamotriginre	-0.362	-1.034	0.310	0.291	-1.33
a GLUTAMATERGIC SYSTEM	Weiser 2012	D-serine	-0.005	-0.345	0.334	0.975	-0.56
a GLUTAMATERGIC SYSTEM	D'Souza 2013	D-serine	0.214	-0.319	0.746	0.431	0.49
a GLUTAMATERGIC SYSTEM	Liu 2013	Minocycline	0.120	-0.318	0.557	0.592	0.16
a GLUTAMATERGIC SYSTEM	Schoemaker 2014 12-16mg	Org 25935	0.043	-0.301	0.386	0.808	-0.26
a GLUTAMATERGIC SYSTEM	Schoemaker 2014 4-8mg	Org 25935	0.041	-0.309	0.391	0.819	-0.26
a GLUTAMATERGIC SYSTEM	Lane 2013	Benzoate	0.281	-0.285	0.847	0.330	0.69
a GLUTAMATERGIC SYSTEM	Kelly 2015	Minocycline	0.214	-0.335	0.763	0.445	0.47
a GLUTAMATERGIC SYSTEM	Lin 2015 s	Sarcosine	0.140	-0.536	0.817	0.684	0.16
a GLUTAMATERGIC SYSTEM	Lin 2015	Sarcosine + Benzoate	0.405	-0.268	1.078	0.238	0.95
a GLUTAMATERGIC SYSTEM	Kantrowitz 2017	D-serine	0.000	-0.719	0.719	1.000	-0.24
a GLUTAMATERGIC SYSTEM			0.085	-0.035	0.205	0.163	
b CHOLINERGIC SYSTEM	Smith (cigarettes) 2002	Nicotine	0.079	-0.429	0.587	0.762	0.30
b CHOLINERGIC SYSTEM	Smith (nasal spray) 2002	Nicotine	0.452	-0.062	0.966	0.085	1.74
b CHOLINERGIC SYSTEM	Tugal 2004	Donepezil	-0.071	-1.116	0.974	0.894	-0.13
b CHOLINERGIC SYSTEM	Lee B.J. 2007	Donepezil	0.146	-0.644	0.936	0.717	0.36
b CHOLINERGIC SYSTEM	Lee S.W. 2007	Galantamine	0.199	-0.576	0.974	0.615	0.50
b CHOLINERGIC SYSTEM	Buchanan 2008	Galantamine	0.435	-0.025	0.894	0.064	1.88
b CHOLINERGIC SYSTEM	Freedman 2008 300mg	DMXB-A	-0.091	-0.583	0.401	0.717	-0.37
b CHOLINERGIC SYSTEM	Freedman 2008 150mg	DMXB-A	0.010	-0.482	0.501	0.969	0.04
b CHOLINERGIC SYSTEM	Keefe 2008	Donepezil	0.060	-0.200	0.320	0.649	0.47
b CHOLINERGIC SYSTEM	Hong 2011	Varenicline	0.034	-0.450	0.518	0.891	0.14
b CHOLINERGIC SYSTEM	Lindenmayer 2011	Galantamine	0.031	-0.646	0.708	0.929	0.09
b CHOLINERGIC SYSTEM	Shim 2012	Varenicline	0.171	-0.237	0.580	0.410	0.83
b CHOLINERGIC SYSTEM	Velligan 2012 35/100mg	AZD3480	-0.319	-0.642	0.003	0.052	-2.00
b CHOLINERGIC SYSTEM	Velligan 2012 20mg	AZD3480	-0.285	-0.596	0.026	0.073	-1.86
b CHOLINERGIC SYSTEM	Velligan 2012 5mg	AZD3480	-0.216	-0.513	0.081	0.153	-1.49
b CHOLINERGIC SYSTEM	Deutsch 2013	nAChR agonist	0.343	-0.252	0.938	0.259	1.14
b CHOLINERGIC SYSTEM	Lieberman 2013	TC-5619	-0.027	-0.341	0.287	0.866	-0.18
b CHOLINERGIC SYSTEM	Umbricht 2014 50mg	RG3487	-0.013	-0.407	0.382	0.951	-0.07
b CHOLINERGIC SYSTEM	Umbricht 2014 15mg	RG3487	-0.475	-0.871	-0.078	0.019	-2.40
b CHOLINERGIC SYSTEM	Umbricht 2014 5mg	RG3487	0.079	-0.318	0.476	0.697	0.39
b CHOLINERGIC SYSTEM	Zhu 2014	Donepezil	0.307	-0.227	0.841	0.259	1.14
b CHOLINERGIC SYSTEM	Keefe 2015 0.9mg	Encenicline	0.387	-0.074	0.849	0.100	1.67
b CHOLINERGIC SYSTEM	Keefe 2015 0.27mg	Encenicline	0.176	-0.281	0.634	0.449	0.76
b CHOLINERGIC SYSTEM	Haig 2016 25mg	ABT-126	0.015	-0.344	0.374	0.934	0.08
b CHOLINERGIC SYSTEM	Haig 2016 10mg	ABT-126	0.014	-0.331	0.358	0.937	0.08
b CHOLINERGIC SYSTEM	Haig 2016b	ABT-126	-0.016	-0.261	0.228	0.896	-0.15
b CHOLINERGIC SYSTEM	Buchanan 2017	Galantamine	0.213	-0.485	0.912	0.549	0.60
b CHOLINERGIC SYSTEM			0.001	-0.077	0.079	0.984	
c SEROTONINERGIC SYSTEM	Poyurovsky 2003	Mianserine	0.431	-0.354	1.216	0.282	1.09
c SEROTONINERGIC SYSTEM	Friedman 2005	Citalopram	0.271	-0.355	0.896	0.397	0.86
c SEROTONINERGIC SYSTEM	Sumiyoshi 2007	Buspirone	0.064	-0.440	0.568	0.804	0.22
c SEROTONINERGIC SYSTEM	Berk 2009	Mirtazapine	-0.355	-1.063	0.353	0.326	-1.08
c SEROTONINERGIC SYSTEM	Piskulic 2009	Buspirone	-0.324	-1.210	0.562	0.473	-0.77
c SEROTONINERGIC SYSTEM	Sternberg 2010	Mirtazapine	-0.063	-0.694	0.568	0.845	-0.25
c SEROTONINERGIC SYSTEM	Morozova 2012	Latrepirdine	-0.150	-0.752	0.451	0.624	-0.58
c SEROTONINERGIC SYSTEM	Morozova 2014	AVN-211	0.115	-0.490	0.720	0.710	0.35
c SEROTONINERGIC SYSTEM			0.014	-0.214	0.241	0.907	
e DOPAMINERGIC SYSTEM	Pietrzak 2010	D-amphetamine	0.188	-0.297	0.673	0.448	
e DOPAMINERGIC SYSTEM			0.188	-0.297	0.673	0.448	
f GABA-ERGIC SYSTEM	Buchanan 2011 16mg	MK-0777	-0.040	-0.688	0.607	0.903	0.04
f GABA-ERGIC SYSTEM	Buchanan 2011 6mg	MK-0777	-0.057	-0.734	0.620	0.868	-0.04
f GABA-ERGIC SYSTEM			-0.048	-0.516	0.420	0.839	
g NORADRENERGIC SYSTEM	Friedman 2008	Atomoxetine	-0.146	-1.102	0.810	0.764	-0.47
g NORADRENERGIC SYSTEM	Kelly 2009	Atomoxetine	-0.144	-0.953	0.664	0.726	-0.58
g NORADRENERGIC SYSTEM	Poyurovsky 2009	Norepinephrine	0.290	-0.380	0.960	0.396	0.94
g NORADRENERGIC SYSTEM			0.055	-0.399	0.509	0.813	
h MISCELLANEOUS	Pierre 2007	Modafinil	-0.319	-1.165	0.526	0.459	-0.61
h MISCELLANEOUS	Kane 2010 150mg	Armodafinil	0.491	-0.280	1.262	0.212	1.43
h MISCELLANEOUS	Kane 2010 100mg	Armodafinil	-0.098	-0.830	0.635	0.794	-0.10
h MISCELLANEOUS	Kane 2010 50mg	Armodafinil	0.255	-0.480	0.990	0.497	0.86
h MISCELLANEOUS	Javitt 2012 30mg	Davunetide	-0.008	-0.639	0.623	0.980	0.17
h MISCELLANEOUS	Javitt 2012 5mg	Davunetide	0.103	-0.537	0.743	0.753	0.52
h MISCELLANEOUS	Kane 2012 250mg	Armodafinil	-0.015	-0.343	0.314	0.930	0.31
h MISCELLANEOUS	Kane 2012 200mg	Armodafinil	-0.240	-0.571	0.092	0.157	-1.16
h MISCELLANEOUS	Kane 2012 150mg	Armodafinil	-0.186	-0.516	0.144	0.269	-0.82
h MISCELLANEOUS	Yi 2012	Rosiglitazone	-0.046	-0.906	0.815	0.917	0.04
h MISCELLANEOUS	Lohr 2013	Modafinil	0.258	-0.518	1.034	0.515	0.82
h MISCELLANEOUS	Huerta-Ramos 2014	Raloxifene	-0.354	-1.296	0.588	0.462	-0.61
h MISCELLANEOUS	Lees 2017	Modafinil	0.023	-0.411	0.457	0.918	0.41
h MISCELLANEOUS			-0.062	-0.205	0.081	0.395	
Overall			0.013	-0.043	0.070	0.647	

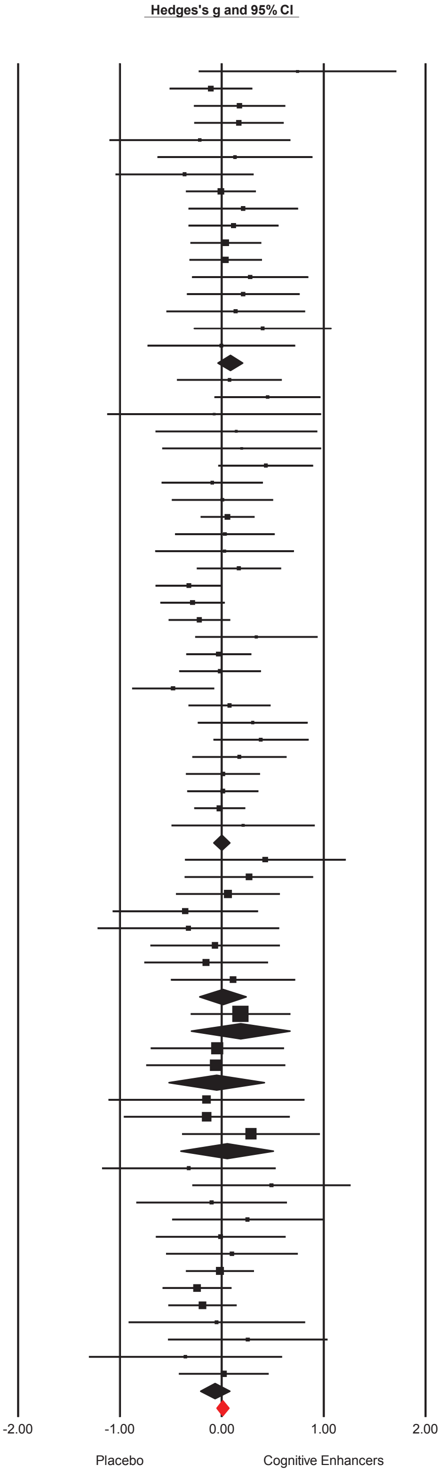

Reasoning and Problem solving

Group by System	Study name	Agent	Hedges's g	Lower limit	Upper limit	p-Value	Std Residual
a GLUTAMATERGIC SYSTEM	Tsai 1998	D-serine	-0.149	-0.858	0.560	0.681	-0.64
a GLUTAMATERGIC SYSTEM	Tsai 1999	D-serine	1.026	0.128	1.923	0.025	2.00
a GLUTAMATERGIC SYSTEM	Goff 2001	Ampakine CX516	0.237	-0.700	1.173	0.620	0.30
a GLUTAMATERGIC SYSTEM	Buchanan 2007 G	Glycine	0.087	-0.367	0.542	0.706	-0.01
a GLUTAMATERGIC SYSTEM	Buchanan 2007	D-Cycloserine	0.087	-0.355	0.530	0.699	-0.01
a GLUTAMATERGIC SYSTEM	Zoccali 2007	Lamotrigine	0.357	-0.188	0.902	0.199	0.90
a GLUTAMATERGIC SYSTEM	Goff 2008	Ampakine CX516	-0.127	-0.529	0.275	0.536	-0.95
a GLUTAMATERGIC SYSTEM	Marx 2009	Pregnenolone	-0.017	-0.897	0.863	0.969	-0.23
a GLUTAMATERGIC SYSTEM	Chengappa 2012	L-carnosine	-0.085	-0.784	0.613	0.811	-0.48
a GLUTAMATERGIC SYSTEM	Weiser 2012	D-serine	0.058	-0.298	0.413	0.751	-0.16
a GLUTAMATERGIC SYSTEM	Vayisoglu 2012	Lamotrigine	0.533	-0.146	1.211	0.124	1.23
a GLUTAMATERGIC SYSTEM	D'Souza 2013	D-serine	-0.010	-0.540	0.521	0.972	-0.35
a GLUTAMATERGIC SYSTEM	Lane 2013	Benzoate	0.131	-0.422	0.684	0.643	0.14
a GLUTAMATERGIC SYSTEM	Liu 2014	Minocycline	0.313	-0.126	0.753	0.163	0.91
a GLUTAMATERGIC SYSTEM	Schoemaker 2014 12-16mg	Org 25935	-0.028	-0.371	0.316	0.875	-0.59
a GLUTAMATERGIC SYSTEM	Schoemaker 2014 4-8mg	Org 25935	-0.029	-0.379	0.321	0.872	-0.58
a GLUTAMATERGIC SYSTEM	Kelly 2015	Minocycline	-0.156	-0.704	0.392	0.578	-0.83
a GLUTAMATERGIC SYSTEM	Lin 2015 S	Sarcosine	0.395	-0.287	1.078	0.256	0.84
a GLUTAMATERGIC SYSTEM	Lin 2015	Sarcosine + Benzoate	0.626	-0.056	1.309	0.072	1.48
a GLUTAMATERGIC SYSTEM	Veerman 2016	Memantine	-0.222	-0.775	0.331	0.432	-1.04
a GLUTAMATERGIC SYSTEM	Kantrowitz 2017	D-serine	-0.013	-0.733	0.706	0.971	-0.27
a GLUTAMATERGIC SYSTEM			0.090	-0.036	0.217	0.162	
b CHOLINERGIC SYSTEM	Tugal 2004	Donepezil	0.559	-0.509	1.627	0.305	1.03
b CHOLINERGIC SYSTEM	Fagerlund 2007	Donepezil	-1.178	-2.404	0.048	0.060	-1.83
b CHOLINERGIC SYSTEM	Kohler 2007	Donepezil	-0.514	-1.332	0.305	0.219	-1.16
b CHOLINERGIC SYSTEM	Akhondzadeh 2008	Donepezil	-0.214	-0.912	0.485	0.548	-0.54
b CHOLINERGIC SYSTEM	Barr 2008	Nicotine	0.509	-0.016	1.034	0.058	1.82
b CHOLINERGIC SYSTEM	Dyer 2008	Galantamine	-1.483	-2.440	-0.526	0.002	-2.94
b CHOLINERGIC SYSTEM	Freedman 2008 300mg	DMXB-A	-0.255	-0.748	0.239	0.312	-0.88
b CHOLINERGIC SYSTEM	Freedman 2008 150mg	DMXB-A	-0.054	-0.546	0.438	0.830	-0.15
b CHOLINERGIC SYSTEM	Keefe 2008	Donepezil	-0.028	-0.288	0.232	0.831	-0.09
b CHOLINERGIC SYSTEM	Shiina 2010	Tropisetron	0.058	-0.608	0.724	0.865	0.20
b CHOLINERGIC SYSTEM	Lindenmayer 2011	Galantamine	0.389	-0.294	1.073	0.264	1.11
b CHOLINERGIC SYSTEM	Shim 2012	Varenicline	-0.113	-0.521	0.295	0.587	-0.43
b CHOLINERGIC SYSTEM	Lieberman 2013	TC-5619	0.282	-0.034	0.598	0.080	1.54
b CHOLINERGIC SYSTEM	Umbricht 2014 50mg	RG3487	-0.062	-0.457	0.333	0.758	-0.21
b CHOLINERGIC SYSTEM	Umbricht 2014 15mg	RG3487	-0.287	-0.680	0.106	0.153	-1.21
b CHOLINERGIC SYSTEM	Umbricht 2014 5mg	RG3487	-0.280	-0.679	0.119	0.169	-1.16
b CHOLINERGIC SYSTEM	Zhu 2014	Donepezil	0.034	-0.501	0.570	0.900	0.16
b CHOLINERGIC SYSTEM	Keefe 2015 0.9mg	Encenicline	0.097	-0.273	0.467	0.608	0.51
b CHOLINERGIC SYSTEM	Keefe 2015 0.27mg	Encenicline	-0.199	-0.568	0.169	0.289	-0.86
b CHOLINERGIC SYSTEM	Haig 2016 25mg	ABT-126	0.115	-0.243	0.474	0.528	0.61
b CHOLINERGIC SYSTEM	Haig 2016 10mg	ABT-126	0.068	-0.277	0.412	0.700	0.40
b CHOLINERGIC SYSTEM	Haig 2016b	ABT-126	0.087	-0.158	0.333	0.485	0.62
b CHOLINERGIC SYSTEM	Buchanan 2017	Galantamine	0.487	-0.220	1.195	0.177	1.33
b CHOLINERGIC SYSTEM			-0.014	-0.120	0.093	0.798	
c SEROTONINERGIC SYSTEM	Sumiyoshi 2001	Tandospirone	0.569	-0.200	1.338	0.147	1.34
c SEROTONINERGIC SYSTEM	Poyurovsky 2003	Mianserine	0.288	-0.491	1.068	0.468	0.63
c SEROTONINERGIC SYSTEM	Friedman 2005	Citalopram	-0.282	-0.908	0.343	0.377	-0.99
c SEROTONINERGIC SYSTEM	Sumiyoshi 2007	Buspirone	-0.089	-0.593	0.415	0.729	-0.48
c SEROTONINERGIC SYSTEM	Akhondzadeh 2008	Ondansetron	0.009	-0.688	0.705	0.981	-0.08
c SEROTONINERGIC SYSTEM	Piskulic 2009	Buspirone	-0.079	-0.959	0.802	0.861	-0.26
c SEROTONINERGIC SYSTEM	Sternberg 2010	Mitazapine	-0.276	-0.910	0.358	0.393	-0.95
c SEROTONINERGIC SYSTEM	Mico 2011	Duloxetine	0.170	-0.438	0.779	0.583	0.42
c SEROTONINERGIC SYSTEM	Morozova 2012	Latrepirdine	0.229	-0.374	0.831	0.457	0.61
c SEROTONINERGIC SYSTEM	Niitsu 2012 50mg	Fluvoxamine	0.160	-0.403	0.723	0.578	0.42
c SEROTONINERGIC SYSTEM	Samadi 2017	Ondansetron	-0.099	-0.722	0.525	0.757	-0.42
c SEROTONINERGIC SYSTEM			0.037	-0.169	0.243	0.726	
e DOPAMINERGIC SYSTEM	Pietrzak 2010	D-amphetamine	0.409	-0.080	0.898	0.101	0.35
e DOPAMINERGIC SYSTEM	Girgis 2016 15mg	DAR-0100A	0.309	-0.361	0.979	0.366	-0.09
e DOPAMINERGIC SYSTEM	Girgis 2016 0.5mg	DAR-0100A	0.243	-0.426	0.912	0.476	-0.31
e DOPAMINERGIC SYSTEM			0.337	-0.030	0.705	0.072	
f GABA-ERGIC SYSTEM	Buchanan 2011 16mg	MK-0777	-0.394	-1.048	0.260	0.238	-0.20
f GABA-ERGIC SYSTEM	Buchanan 2011 6mg	MK-0777	-0.294	-0.974	0.387	0.398	0.20
f GABA-ERGIC SYSTEM			-0.346	-0.845	0.154	0.175	
g NORADRENERGIC SYSTEM	Friedman 2008	Atomoxetine	0.353	-0.610	1.316	0.472	0.61
g NORADRENERGIC SYSTEM	Kelly 2009	Atomoxetine	0.275	-0.536	1.087	0.506	0.54
g NORADRENERGIC SYSTEM	Poyurovsky 2009	Norepinephrine	-0.188	-0.855	0.480	0.582	-1.02
g NORADRENERGIC SYSTEM			0.084	-0.392	0.559	0.730	
h MISCELLANEOUS	Kane 2010 150mg	Armodafinil	-0.014	-0.773	0.744	0.970	0.19
h MISCELLANEOUS	Kane 2010 100mg	Armodafinil	-0.038	-0.770	0.694	0.920	0.13
h MISCELLANEOUS	Kane 2010 50mg	Armodafinil	0.393	-0.347	1.132	0.298	1.25
h MISCELLANEOUS	Bobo 2011	Armodafinil	-1.459	-2.032	-0.886	0.000	-4.51
h MISCELLANEOUS	Javitt 2012 30mg	Davunetide	-0.030	-0.661	0.601	0.925	0.18
h MISCELLANEOUS	Javitt 2012 5mg	Davunetide	0.134	-0.506	0.775	0.682	0.66
h MISCELLANEOUS	Kane 2012 250mg	Armodafinil	-0.026	-0.354	0.303	0.879	0.34
h MISCELLANEOUS	Kane 2012 200mg	Armodafinil	0.032	-0.298	0.363	0.849	0.64
h MISCELLANEOUS	Kane 2012 150mg	Armodafinil	-0.048	-0.378	0.281	0.775	0.22
h MISCELLANEOUS	Yi 2012	Rosiglitazone	-0.096	-0.956	0.765	0.828	-0.02
h MISCELLANEOUS	Lees 2017	Modafinil	0.059	-0.375	0.493	0.791	0.62
h MISCELLANEOUS			-0.088	-0.256	0.079	0.300	
Overall			0.016	-0.051	0.082	0.639	

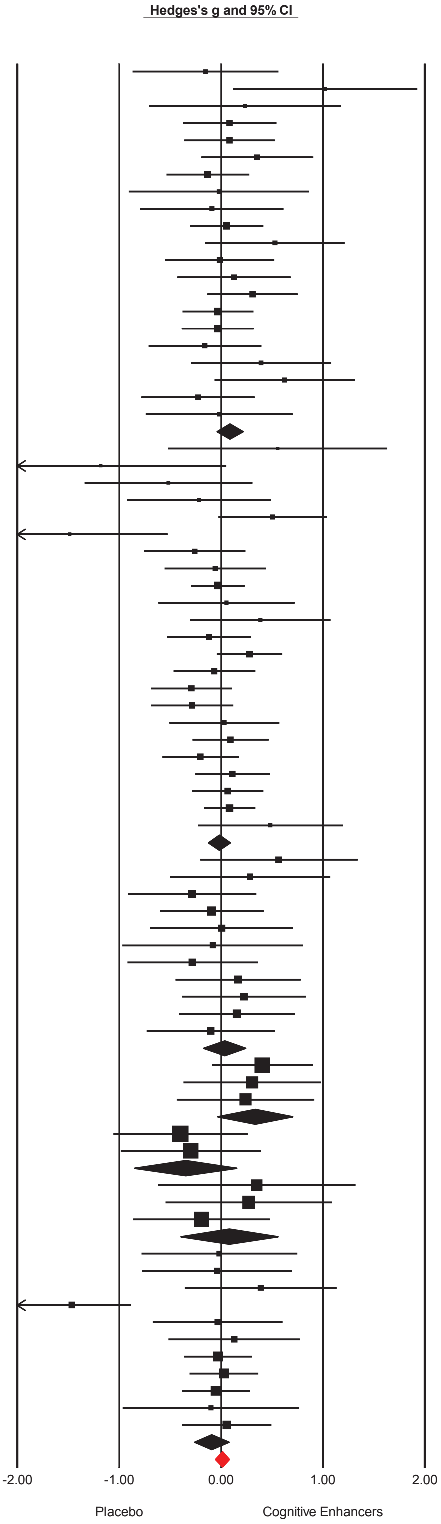

Verbal Fluency

Group by System	Study name	Agent	Hedges's g	Lower limit	Upper limit	p-Value	Std Residual
			**Statistics for each study**				
a GLUTAMATERGIC SYSTEM	Goff 2001	Ampakine CX516	0.375	-0.567	1.316	0.435	0.95
a GLUTAMATERGIC SYSTEM	Buchanan 2007	g Glycine	-0.170	-0.619	0.279	0.458	-0.51
a GLUTAMATERGIC SYSTEM	Buchanan 2007	D-Cycloserine	-0.198	-0.639	0.242	0.377	-0.67
a GLUTAMATERGIC SYSTEM	Lee 2012	Memantine	0.024	-0.730	0.777	0.950	0.25
a GLUTAMATERGIC SYSTEM	Weiser 2012	D-serine	-0.007	-0.351	0.337	0.969	0.45
a GLUTAMATERGIC SYSTEM			-0.068	-0.284	0.148	0.537	
b CHOLINERGIC SYSTEM	Tugal 2004	Donepezil	-0.612	-1.685	0.461	0.264	-0.88
b CHOLINERGIC SYSTEM	Schubert 2006	Galatamine	0.534	-0.476	1.545	0.300	1.33
b CHOLINERGIC SYSTEM	Lee B.J. 2007	Donepezil	0.739	-0.078	1.556	0.076	2.18
b CHOLINERGIC SYSTEM	Lee S.W. 2007	Galatamine	0.000	-0.773	0.773	1.000	0.36
b CHOLINERGIC SYSTEM	Keefe 2008	Donepezil	-0.315	-0.576	-0.053	0.018	-2.13
b CHOLINERGIC SYSTEM	Zhang 2012 20mg	Tropisetron	0.031	-0.855	0.916	0.946	0.38
b CHOLINERGIC SYSTEM	Zhang 2012 10mg	Tropisetron	-0.246	-1.089	0.597	0.567	-0.26
b CHOLINERGIC SYSTEM	Zhang 2012 5mg	Tropisetron	0.380	-0.468	1.227	0.380	1.23
b CHOLINERGIC SYSTEM			-0.138	-0.344	0.067	0.187	
c SEROTONINERGIC SYSTEM	Friedman 2005	Citalopram	0.115	-0.508	0.738	0.718	0.47
c SEROTONINERGIC SYSTEM	Sumiyoshi 2007	Buspirone	-0.068	-0.572	0.436	0.791	-0.22
c SEROTONINERGIC SYSTEM	Berk 2009	Mitazapine	-0.454	-1.121	0.212	0.182	-1.40
c SEROTONINERGIC SYSTEM	Sternberg 2010	Mitazapine	0.055	-0.576	0.686	0.863	0.26
c SEROTONINERGIC SYSTEM	Mico 2011	Duloxetine	0.219	-0.391	0.828	0.482	0.85
c SEROTONINERGIC SYSTEM			-0.019	-0.287	0.248	0.888	
f GABA-ERGIC SYSTEM	Lewis 2008	MK-0777	-0.240	-1.216	0.736	0.630	
f GABA-ERGIC SYSTEM			-0.240	-1.216	0.736	0.630	
g NORADRENERGIC SYSTEM	Friedman 2008	Atomoxetine	0.288	-0.672	1.249	0.556	0.11
g NORADRENERGIC SYSTEM	Kelly 2009	Atomoxetine	0.220	-0.590	1.030	0.594	-0.11
g NORADRENERGIC SYSTEM			0.248	-0.371	0.868	0.432	
h MISCELLANEOUS	Sevy 2005	Modafinil	0.140	-0.701	0.980	0.745	0.18
h MISCELLANEOUS	Goff 2009 100mg	Sildenafil	-0.192	-0.850	0.466	0.567	-0.86
h MISCELLANEOUS	Goff 2009 50mg	Sildenafil	-0.328	-0.989	0.333	0.331	-1.30
h MISCELLANEOUS	Bobo 2011	Armodafinil	0.531	0.014	1.048	0.044	2.08
h MISCELLANEOUS	Yi 2012	Rosiglitazone	0.215	-0.648	1.078	0.625	0.35
h MISCELLANEOUS	Huerta-Ramos 2014	Raloxifene	-0.225	-0.974	0.524	0.556	-0.83
h MISCELLANEOUS			0.068	-0.210	0.347	0.632	
Overall			-0.049	-0.164	0.066	0.400	

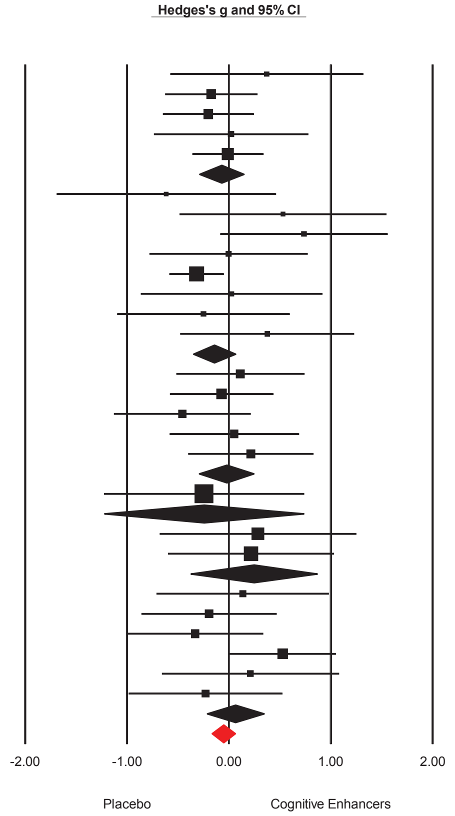

Verbal learning and memory

Group by System	Study name	Hedges's g	Lower limit	Upper limit	p-Value	Std Residual
a GLUTAMATERGIC SYSTEM	Goff 2001	0.921	-0.059	1.902	0.066	1.66
a GLUTAMATERGIC SYSTEM	Buchanan 2007 G	-0.144	-0.592	0.305	0.530	-0.97
a GLUTAMATERGIC SYSTEM	Buchanan 2007	0.129	-0.311	0.569	0.566	0.19
a GLUTAMATERGIC SYSTEM	Marx 2009	0.455	-0.438	1.347	0.318	0.81
a GLUTAMATERGIC SYSTEM	Chengappa 2012	0.012	-0.501	0.525	0.964	-0.27
a GLUTAMATERGIC SYSTEM	Lee 2012	0.027	-0.726	0.781	0.944	-0.15
a GLUTAMATERGIC SYSTEM	Weiser 2012	0.090	-0.262	0.442	0.615	0.03
a GLUTAMATERGIC SYSTEM	Vayisoglu 2012	-0.043	-0.709	0.623	0.900	-0.37
a GLUTAMATERGIC SYSTEM	D'Souza 2013	0.145	-0.386	0.676	0.593	0.22
a GLUTAMATERGIC SYSTEM	Lane 2013	0.316	-0.240	0.871	0.265	0.80
a GLUTAMATERGIC SYSTEM	Liu 2014	0.022	-0.415	0.459	0.921	-0.27
a GLUTAMATERGIC SYSTEM	Schoemaker 2014 12-16mg	0.144	-0.200	0.488	0.412	0.32
a GLUTAMATERGIC SYSTEM	Schoemaker 2014 4-8mg	0.002	-0.347	0.352	0.989	-0.44
a GLUTAMATERGIC SYSTEM	Kelly 2015	-0.194	-0.743	0.355	0.489	-0.98
a GLUTAMATERGIC SYSTEM	Lin 2015 S	-0.208	-0.803	0.387	0.493	-0.95
a GLUTAMATERGIC SYSTEM	Lin 2015	0.271	-0.325	0.868	0.373	0.60
a GLUTAMATERGIC SYSTEM	Veerman 2016	0.239	-0.314	0.792	0.397	0.53
a GLUTAMATERGIC SYSTEM	Kantrowitz 2017	0.265	-0.458	0.987	0.473	0.48
a GLUTAMATERGIC SYSTEM		0.085	-0.042	0.212	0.188	
b CHOLINERGIC SYSTEM	Friedman 2002 10mg	-0.024	-0.831	0.782	0.953	-0.23
b CHOLINERGIC SYSTEM	Friedman 2002 5mg	-0.077	-0.827	0.674	0.842	-0.38
b CHOLINERGIC SYSTEM	Tugal 2004	-0.314	-1.366	0.738	0.559	-0.71
b CHOLINERGIC SYSTEM	Schubert 2006	0.481	-0.526	1.488	0.349	0.79
b CHOLINERGIC SYSTEM	Fagerlund 2007	-1.617	-2.928	-0.306	0.016	-2.51
b CHOLINERGIC SYSTEM	Kohler 2007	0.095	-0.710	0.899	0.818	0.05
b CHOLINERGIC SYSTEM	Lee B.J. 2007	0.889	0.060	1.718	0.036	1.90
b CHOLINERGIC SYSTEM	Lee S.W. 2007	0.228	-0.548	1.003	0.565	0.39
b CHOLINERGIC SYSTEM	Akhondzadeh 2008	0.968	0.230	1.706	0.010	2.34
b CHOLINERGIC SYSTEM	Buchanan 2008	0.299	-0.158	0.756	0.200	0.93
b CHOLINERGIC SYSTEM	Freedman 2008 300mg	-0.086	-0.578	0.406	0.732	-0.61
b CHOLINERGIC SYSTEM	Freedman 2008 150mg	-0.312	-0.806	0.183	0.217	-1.47
b CHOLINERGIC SYSTEM	Keefe 2008	-0.069	-0.329	0.191	0.604	-0.95
b CHOLINERGIC SYSTEM	Zhang 2012 20mg	0.718	-0.198	1.634	0.125	1.36
b CHOLINERGIC SYSTEM	Zhang 2012 10mg	0.679	-0.187	1.544	0.124	1.36
b CHOLINERGIC SYSTEM	Zhang 2012 5mg	0.188	-0.653	1.030	0.661	0.27
b CHOLINERGIC SYSTEM	Deutsch 2013	0.519	-0.082	1.120	0.090	1.42
b CHOLINERGIC SYSTEM	Lieberman 2013	0.149	-0.166	0.464	0.353	0.44
b CHOLINERGIC SYSTEM	Umbricht 2014 50mg	-0.338	-0.736	0.060	0.096	-1.91
b CHOLINERGIC SYSTEM	Umbricht 2014 15mg	-0.327	-0.721	0.066	0.103	-1.88
b CHOLINERGIC SYSTEM	Umbricht 2014 5mg	-0.337	-0.737	0.063	0.098	-1.90
b CHOLINERGIC SYSTEM	Zhu 2014	0.293	-0.245	0.832	0.285	0.78
b CHOLINERGIC SYSTEM	Keefe 2015 0.9mg	0.422	-0.017	0.861	0.060	1.49
b CHOLINERGIC SYSTEM	Keefe 2015 0.27mg	0.368	-0.058	0.793	0.091	1.29
b CHOLINERGIC SYSTEM	Haig 2016 25mg	0.147	-0.212	0.507	0.421	0.38
b CHOLINERGIC SYSTEM	Haig 2016 10mg	0.073	-0.272	0.417	0.680	-0.00
b CHOLINERGIC SYSTEM	Haig 2016b	0.028	-0.216	0.273	0.822	-0.31
b CHOLINERGIC SYSTEM	Buchanan 2017	0.252	-0.447	0.951	0.480	0.49
b CHOLINERGIC SYSTEM		0.073	-0.026	0.171	0.147	
c SEROTONINERGIC SYSTEM	Sumiyoshi 2001	0.565	-0.204	1.334	0.150	1.47
c SEROTONINERGIC SYSTEM	Friedman 2005	0.307	-0.319	0.934	0.337	0.99
c SEROTONINERGIC SYSTEM	Sumiyoshi 2007	-0.247	-0.752	0.259	0.339	-1.03
c SEROTONINERGIC SYSTEM	Akhondzadeh 2009	0.326	-0.376	1.027	0.363	0.93
c SEROTONINERGIC SYSTEM	Berk 2009	-0.375	-1.039	0.288	0.267	-1.16
c SEROTONINERGIC SYSTEM	Piskulic 2009	-0.032	-0.912	0.848	0.943	-0.08
c SEROTONINERGIC SYSTEM	Sternberg 2010	0.249	-0.385	0.882	0.442	0.79
c SEROTONINERGIC SYSTEM	Morozova 2012	-0.431	-1.039	0.177	0.165	-1.46
c SEROTONINERGIC SYSTEM		0.003	-0.235	0.241	0.980	
f GABA-ERGIC SYSTEM	Lewis 2008	-0.009	-0.981	0.964	0.986	0.53
f GABA-ERGIC SYSTEM	Buchanan 2011 16mg	-0.402	-1.057	0.252	0.229	-0.58
f GABA-ERGIC SYSTEM	Buchanan 2011 6mg	-0.204	-0.882	0.475	0.556	0.16
f GABA-ERGIC SYSTEM		-0.248	-0.684	0.188	0.264	
g NORADRENERGIC SYSTEM	Friedman 2008	0.205	-0.753	1.162	0.675	-0.29
g NORADRENERGIC SYSTEM	Kelly 2009	0.392	-0.424	1.207	0.347	0.29
g NORADRENERGIC SYSTEM		0.313	-0.320	0.945	0.333	
h MISCELLANEOUS	Sevy 2005	0.411	-0.438	1.260	0.343	1.18
h MISCELLANEOUS	Pierre 2007	0.053	-0.787	0.892	0.902	0.36
h MISCELLANEOUS	Lees 2017	0.069	-0.365	0.503	0.756	0.75
h MISCELLANEOUS	Goff 2008 100mg	-0.202	-0.860	0.456	0.548	-0.30
h MISCELLANEOUS	Goff 2008 50mg	-0.071	-0.728	0.585	0.831	0.09
h MISCELLANEOUS	Kane 2010 150mg	0.476	-0.294	1.246	0.226	1.46
h MISCELLANEOUS	Kane 2010 100mg	0.260	-0.475	0.995	0.488	0.96
h MISCELLANEOUS	Kane 2010 50mg	0.159	-0.575	0.892	0.671	0.69
h MISCELLANEOUS	Javitt 2012 30mg	-0.296	-0.931	0.338	0.360	-0.60
h MISCELLANEOUS	Javitt 2012 5mg	-0.653	-1.310	0.005	0.052	-1.63
h MISCELLANEOUS	Kane 2012 250mg	-0.060	-0.389	0.268	0.719	0.23
h MISCELLANEOUS	Kane 2012 200mg	-0.365	-0.698	-0.031	0.032	-1.51
h MISCELLANEOUS	Kane 2012 150mg	-0.087	-0.416	0.243	0.606	0.08
h MISCELLANEOUS	Yi 2012	-0.162	-1.024	0.699	0.712	-0.14
h MISCELLANEOUS	Huerta-Ramos 2014	-0.285	-1.035	0.466	0.457	-0.48
h MISCELLANEOUS		-0.101	-0.248	0.047	0.180	
Overall		0.032	-0.033	0.097	0.327	

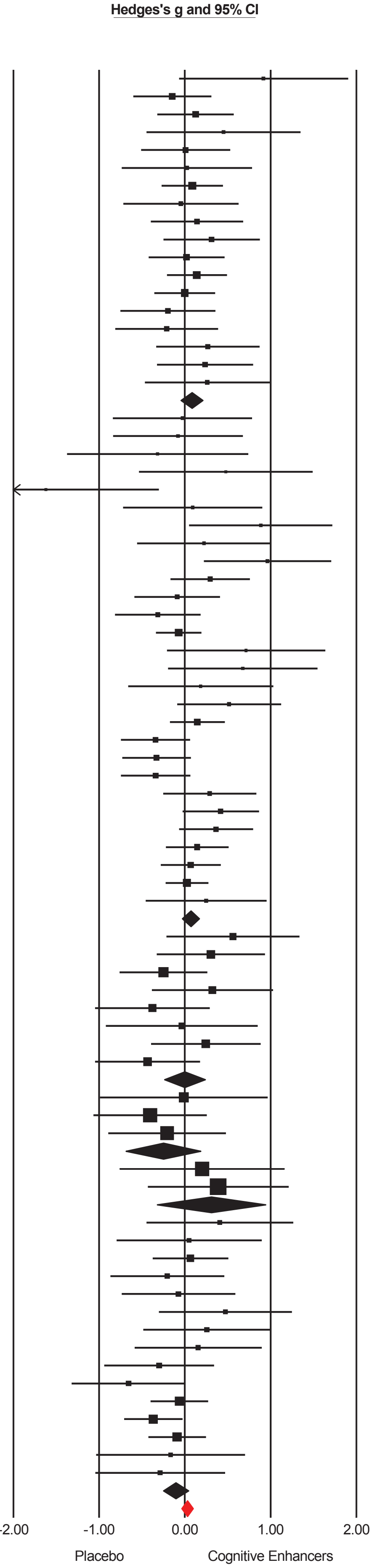

Visual learning and memory

Group by System	Study name	Agent	Hedges's g	Lower limit	Upper limit	p-Value	Std Residual
a GLUTAMATERGIC SYSTEM	Goff 2001	Ampakine CX516	0.568	-0.383	1.520	0.242	1.02
a GLUTAMATERGIC SYSTEM	Silver 2005	Amantadine	0.083	-0.706	0.872	0.836	0.02
a GLUTAMATERGIC SYSTEM	Buchanan 2007 G	Glycine	-0.199	-0.654	0.256	0.391	-1.19
a GLUTAMATERGIC SYSTEM	Buchanan 2007	D-Cycloserine	-0.225	-0.669	0.219	0.321	-1.33
a GLUTAMATERGIC SYSTEM	Goff 2008	Ampakine CX516	-0.068	-0.469	0.334	0.742	-0.70
a GLUTAMATERGIC SYSTEM	Marx 2009	Pregnenolone	-0.228	-1.111	0.655	0.613	-0.67
a GLUTAMATERGIC SYSTEM	Levkovitz 2010	Minocycline	0.629	-0.238	1.495	0.155	1.26
a GLUTAMATERGIC SYSTEM	Lee 2012	Memantine	0.027	-0.726	0.781	0.943	-0.12
a GLUTAMATERGIC SYSTEM	Weiser 2012	D-serine	0.095	-0.246	0.436	0.586	0.12
a GLUTAMATERGIC SYSTEM	Lane 2013	Benzoate	0.495	-0.071	1.061	0.086	1.47
a GLUTAMATERGIC SYSTEM	Liu 2014	Minocycline	0.129	-0.308	0.566	0.563	0.25
a GLUTAMATERGIC SYSTEM	Shoemaker 2014 12-16mg	Org 25935	-0.061	-0.404	0.283	0.729	-0.78
a GLUTAMATERGIC SYSTEM	Shoemaker 2014 4-8mg	Org 25935	0.168	-0.182	0.519	0.346	0.54
a GLUTAMATERGIC SYSTEM	Kelly 2015	Minocycline	0.050	-0.498	0.597	0.859	-0.09
a GLUTAMATERGIC SYSTEM	Lin 2015 S	Sarcosine	0.041	-0.552	0.635	0.892	-0.11
a GLUTAMATERGIC SYSTEM	Lin 2015	Sarcosine + Benzoate	0.410	-0.190	1.010	0.181	1.11
a GLUTAMATERGIC SYSTEM	Veerman 2016	Memantine	0.174	-0.378	0.726	0.537	0.36
a GLUTAMATERGIC SYSTEM	Kantrowitz 2017	D-serine	0.211	-0.510	0.933	0.566	0.38
a GLUTAMATERGIC SYSTEM			0.074	-0.048	0.196	0.236	
b CHOLINERGIC SYSTEM	Smith 2002	Nicotine	0.213	-0.280	0.706	0.398	0.71
b CHOLINERGIC SYSTEM	Tugal 2004	Donepezil	-1.130	-2.132	-0.127	0.027	-2.27
b CHOLINERGIC SYSTEM	Schubert 2006	Galatamine	0.007	-0.984	0.998	0.989	-0.05
b CHOLINERGIC SYSTEM	Kohler 2007	Donepezil	-0.014	-0.818	0.790	0.974	-0.12
b CHOLINERGIC SYSTEM	Lee B.J. 2007	Donepezil	0.713	-0.102	1.528	0.086	1.63
b CHOLINERGIC SYSTEM	Lee S.W. 2007	Galatamine	0.110	-0.663	0.884	0.780	0.19
b CHOLINERGIC SYSTEM	Akhondzadeh 2008	Donepezil	0.015	-0.682	0.711	0.967	-0.05
b CHOLINERGIC SYSTEM	Buchanan 2008	Galatamine	-0.049	-0.503	0.406	0.834	-0.35
b CHOLINERGIC SYSTEM	Freedman 2008 300mg	DMXB-A	-0.098	-0.590	0.394	0.697	-0.52
b CHOLINERGIC SYSTEM	Freedman 2008 150mg	DMXB-A	0.040	-0.452	0.531	0.874	0.02
b CHOLINERGIC SYSTEM	Keefe 2008	Donepezil	0.047	-0.213	0.307	0.722	0.10
b CHOLINERGIC SYSTEM	Shiina 2010	Tropisetron	-0.530	-1.208	0.148	0.126	-1.62
b CHOLINERGIC SYSTEM	Velligan 2012 35/100mg	AZD3480	-0.142	-0.463	0.179	0.387	-1.06
b CHOLINERGIC SYSTEM	Velligan 2012 20mg	AZD3480	-0.008	-0.317	0.302	0.962	-0.26
b CHOLINERGIC SYSTEM	Velligan 2012 5mg	AZD3480	-0.161	-0.457	0.135	0.287	-1.27
b CHOLINERGIC SYSTEM	Zhang 2012 20mg	Tropisetron	0.575	-0.330	1.480	0.213	1.17
b CHOLINERGIC SYSTEM	Zhang 2012 10mg	Tropisetron	-0.030	-0.869	0.810	0.944	-0.15
b CHOLINERGIC SYSTEM	Zhang 2012 5mg	Tropisetron	0.332	-0.514	1.177	0.442	0.69
b CHOLINERGIC SYSTEM	Deutsch 2013	nAChR agonist	0.395	-0.202	0.991	0.195	1.18
b CHOLINERGIC SYSTEM	Lieberman 2013	TC-5619	-0.161	-0.476	0.154	0.317	-1.19
b CHOLINERGIC SYSTEM	Umbricht 2014 50mg	RG3487	0.209	-0.187	0.605	0.302	0.86
b CHOLINERGIC SYSTEM	Umbricht 2014 15mg	RG3487	0.413	0.018	0.808	0.041	1.86
b CHOLINERGIC SYSTEM	Umbricht 2014 5mg	RG3487	0.189	-0.209	0.587	0.352	0.76
b CHOLINERGIC SYSTEM	Zhu 2014	Donepezil	0.805	0.248	1.362	0.005	2.70
b CHOLINERGIC SYSTEM	Keefe 2015 0.9mg	Encenicline	0.051	-0.383	0.485	0.819	0.08
b CHOLINERGIC SYSTEM	Keefe 2015 0.27mg	Encenicline	0.211	-0.212	0.634	0.329	0.81
b CHOLINERGIC SYSTEM	Haig 2016 25mg	ABT-126	0.031	-0.327	0.390	0.864	-0.01
b CHOLINERGIC SYSTEM	Haig 2016 10mg	ABT-126	-0.015	-0.359	0.330	0.934	-0.27
b CHOLINERGIC SYSTEM	Haig 2016b	ABT-126	-0.075	-0.320	0.170	0.547	-0.85
b CHOLINERGIC SYSTEM	Buchanan 2017	Galatamine	-0.258	-0.958	0.441	0.469	-0.82
b CHOLINERGIC SYSTEM			0.034	-0.048	0.116	0.419	
c SEROTONINERGIC SYSTEM	Poyurovsky 2003	Mianserine	1.014	0.187	1.841	0.016	2.24
c SEROTONINERGIC SYSTEM	Akhondzadeh 2009	Ondansetron	0.648	-0.068	1.363	0.076	1.56
c SEROTONINERGIC SYSTEM	Sternberg 2010	Mitazapine	-0.081	-0.712	0.550	0.802	-0.71
c SEROTONINERGIC SYSTEM	Morozova 2012	Latrepirdine	0.116	-0.485	0.718	0.704	-0.03
c SEROTONINERGIC SYSTEM	Niitsu 2012 50mg	Fluvoxamine	-0.467	-1.037	0.103	0.109	-2.33
c SEROTONINERGIC SYSTEM			0.124	-0.171	0.419	0.409	
e DOPAMINERGIC SYSTEM	Pietrzak 2010	D-amphetamine	0.292	-0.194	0.779	0.239	
e DOPAMINERGIC SYSTEM			0.292	-0.204	0.789	0.248	
f GABA-ERGIC SYSTEM	Lewis 2008	MK-0777	-0.461	-1.448	0.525	0.359	-0.18
f GABA-ERGIC SYSTEM	Buchanan 2011 16mg	MK-0777	-0.386	-1.040	0.268	0.247	-0.02
f GABA-ERGIC SYSTEM	Buchanan 2011 6mg	MK-0777	-0.337	-1.019	0.344	0.332	0.16
f GABA-ERGIC SYSTEM			-0.381	-0.811	0.049	0.082	
g NORADRENERGIC SYSTEM	Kelly 2009	Atomoxetine	-0.086	-0.894	0.722	0.834	-0.08
g NORADRENERGIC SYSTEM	Poyurovsky 2009	Norepinephrine	-0.043	-0.710	0.623	0.898	0.08
g NORADRENERGIC SYSTEM			-0.061	-0.580	0.458	0.818	
h MISCELLANEOUS	Sevy 2005	Modafinil	-0.413	-1.262	0.436	0.341	-1.18
h MISCELLANEOUS	Kane 2010 150mg	Armodafinil	0.125	-0.634	0.885	0.747	0.12
h MISCELLANEOUS	Kane 2010 100mg	Armodafinil	0.331	-0.406	1.068	0.379	0.70
h MISCELLANEOUS	Kane 2010 50 mg	Armodafinil	0.444	-0.298	1.186	0.241	1.01
h MISCELLANEOUS	Javitt 2012 30mg	Davunetide	0.106	-0.526	0.737	0.743	0.09
h MISCELLANEOUS	Javitt 2012 5mg	Davunetide	0.448	-0.200	1.096	0.176	1.19
h MISCELLANEOUS	Lees 2017	Modafinil	-0.204	-0.639	0.231	0.358	-1.50
h MISCELLANEOUS			0.080	-0.166	0.326	0.525	
Overall			0.045	-0.017	0.108	0.155	

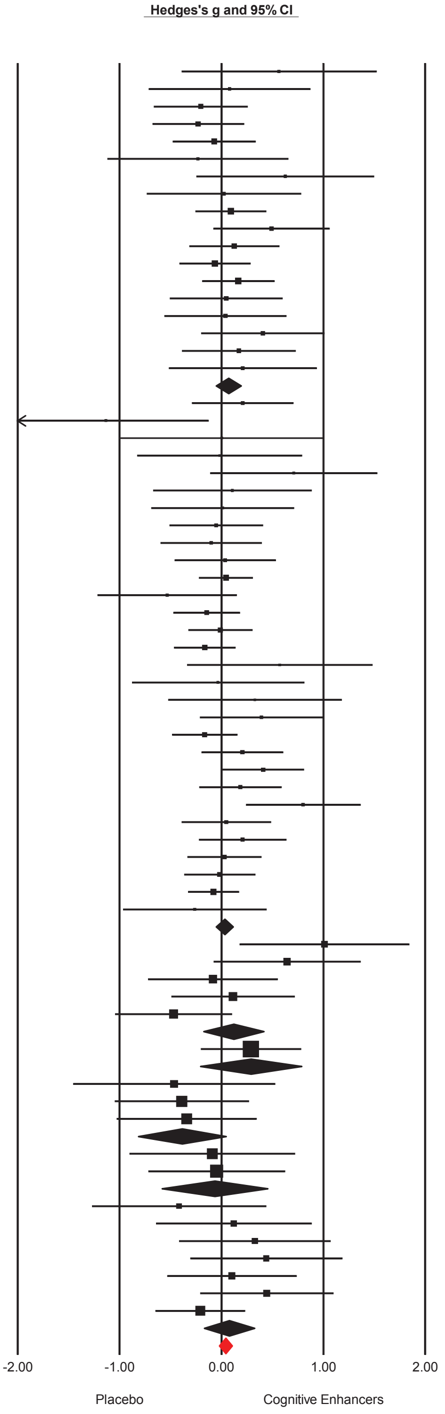

Working memory

Group by System	Study name	Agent	Hedges's g	Lower limit	Upper limit	p-Value	Std Residual
a GLUTAMATERGIC SYSTEM	Duncan 2004	D-Cycloserine	-0.692	-1.678	0.295	0.169	-1.61
a GLUTAMATERGIC SYSTEM	Silver 2005	Amantadine	0.568	-0.238	1.373	0.167	1.03
a GLUTAMATERGIC SYSTEM	Buchanan 2007 G	Glycine	-0.095	-0.583	0.393	0.704	-0.86
a GLUTAMATERGIC SYSTEM	Buchanan 2007	D-Cycloserine	-0.009	-0.476	0.458	0.969	-0.56
a GLUTAMATERGIC SYSTEM	Goff 2008	Ampakine CX516	0.069	-0.332	0.471	0.735	-0.29
a GLUTAMATERGIC SYSTEM	Marx 2009	Pregnenolone	0.110	-0.771	0.991	0.807	-0.05
a GLUTAMATERGIC SYSTEM	Levkovitz 2010	Minocycline	1.542	0.576	2.507	0.002	2.81
a GLUTAMATERGIC SYSTEM	Chengappa 2012	L-carnosine	0.521	0.004	1.038	0.048	1.39
a GLUTAMATERGIC SYSTEM	Vayisoglu 2012	Lamotrigine	-0.000	-0.666	0.666	1.000	-0.38
a GLUTAMATERGIC SYSTEM	Weiser 2012	D-serine	0.149	-0.198	0.497	0.400	0.08
a GLUTAMATERGIC SYSTEM	D'Souza 2013	D-serine	0.147	-0.384	0.679	0.587	0.05
a GLUTAMATERGIC SYSTEM	Lane 2013	Benzoate	0.028	-0.546	0.603	0.923	-0.34
a GLUTAMATERGIC SYSTEM	Liu 2014	Minocycline	0.115	-0.322	0.553	0.605	-0.07
a GLUTAMATERGIC SYSTEM	Schoemaker 2014 12-16mg	Org 25935	-0.077	-0.421	0.266	0.659	-1.07
a GLUTAMATERGIC SYSTEM	Schoemaker 2014 4-8mg	Org 25935	0.024	-0.326	0.374	0.892	-0.55
a GLUTAMATERGIC SYSTEM	Kelly 2015	Minocycline	0.526	-0.031	1.083	0.064	1.32
a GLUTAMATERGIC SYSTEM	Lin 2015 S	Sarcosine	0.081	-0.513	0.674	0.790	-0.17
a GLUTAMATERGIC SYSTEM	Lin 2015	Sarcosine + Benzoate	0.356	-0.242	0.955	0.243	0.70
a GLUTAMATERGIC SYSTEM	Veerman 2016	Memantine	0.182	-0.370	0.735	0.518	0.17
a GLUTAMATERGIC SYSTEM	Kantrowitz 2017	D-serine	0.090	-0.629	0.810	0.806	-0.11
a GLUTAMATERGIC SYSTEM			0.133	0.006	0.260	0.040	
b CHOLINERGIC SYSTEM	Tugal 2004	Donepezil	0.128	-0.918	1.174	0.810	0.16
b CHOLINERGIC SYSTEM	Kumari 2006	Rivastigmine	0.629	-0.215	1.473	0.144	1.33
b CHOLINERGIC SYSTEM	Schubert 2006	Galantamine	1.314	0.210	2.418	0.020	2.22
b CHOLINERGIC SYSTEM	Smith 2006	Nicotine	0.610	0.062	1.158	0.029	1.91
b CHOLINERGIC SYSTEM	Lee B.J. 2007	Donepezil	0.828	0.004	1.652	0.049	1.82
b CHOLINERGIC SYSTEM	Lee S.W. 2007	Galantamine	-0.336	-1.114	0.443	0.398	-0.92
b CHOLINERGIC SYSTEM	Akhondzadeh 2008	Donepezil	0.374	-0.329	1.077	0.297	0.89
b CHOLINERGIC SYSTEM	Barr 2008	Nicotine	-0.172	-0.690	0.345	0.514	-0.75
b CHOLINERGIC SYSTEM	Buchanan 2008	Galantamine	0.168	-0.288	0.623	0.470	0.50
b CHOLINERGIC SYSTEM	Dyer 2008	Galantamine	-0.182	-1.023	0.660	0.672	-0.51
b CHOLINERGIC SYSTEM	Freedman 2008 300mg	DMXB-A	-0.067	-0.558	0.425	0.790	-0.40
b CHOLINERGIC SYSTEM	Freedman 2008 150mg	DMXB-A	0.034	-0.457	0.526	0.891	-0.03
b CHOLINERGIC SYSTEM	Keefe 2008	Donepezil	-0.082	-0.342	0.178	0.538	-0.74
b CHOLINERGIC SYSTEM	Shiina 2010	Tropisetron	-0.104	-0.771	0.562	0.759	-0.41
b CHOLINERGIC SYSTEM	Lindenmayer 2011	Galantamine	0.147	-0.531	0.825	0.671	0.29
b CHOLINERGIC SYSTEM	Shim 2012	Varenicline	-0.183	-0.593	0.228	0.383	-0.96
b CHOLINERGIC SYSTEM	Velligan 2012 35/100mg	AZD3480	-0.218	-0.539	0.103	0.184	-1.34
b CHOLINERGIC SYSTEM	Velligan 2012 20mg	AZD3480	-0.098	-0.408	0.211	0.533	-0.74
b CHOLINERGIC SYSTEM	Velligan 2012 5mg	AZD3480	-0.110	-0.406	0.186	0.466	-0.83
b CHOLINERGIC SYSTEM	Zhang 2012 20mg	Tropisetron	-0.076	-0.962	0.810	0.866	-0.25
b CHOLINERGIC SYSTEM	Zhang 2012 10mg	Tropisetron	0.051	-0.789	0.891	0.905	0.02
b CHOLINERGIC SYSTEM	Zhang 2012 5mg	Tropisetron	0.522	-0.333	1.376	0.232	1.07
b CHOLINERGIC SYSTEM	Deutsch 2013	nAChR agonist	-0.146	-0.738	0.446	0.629	-0.59
b CHOLINERGIC SYSTEM	Lieberman 2013	TC-5619	0.279	-0.037	0.595	0.083	1.25
b CHOLINERGIC SYSTEM	Umbricht 2014 50mg	RG3487	-0.052	-0.447	0.343	0.797	-0.41
b CHOLINERGIC SYSTEM	Umbricht 2014 15mg	RG3487	-0.229	-0.621	0.163	0.252	-1.20
b CHOLINERGIC SYSTEM	Umbricht 2014 5mg	RG3487	0.061	-0.336	0.458	0.765	0.09
b CHOLINERGIC SYSTEM	Zhu 2014	Donepezil	0.934	0.369	1.499	0.001	2.92
b CHOLINERGIC SYSTEM	Keefe 2015 0.9mg	Encenicline	0.045	-0.389	0.479	0.839	0.02
b CHOLINERGIC SYSTEM	Keefe 2015 0.27mg	Encenicline	0.134	-0.288	0.557	0.534	0.39
b CHOLINERGIC SYSTEM	Haig 2016 25mg	ABT-126	0.140	-0.219	0.499	0.446	0.47
b CHOLINERGIC SYSTEM	Haig 2016 10mg	ABT-126	0.046	-0.298	0.391	0.794	0.02
b CHOLINERGIC SYSTEM	Haig 2016b	ABT-126	0.011	-0.234	0.255	0.933	-0.19
b CHOLINERGIC SYSTEM	Buchanan 2017	Galantamine	-0.408	-1.112	0.296	0.256	-1.20
b CHOLINERGIC SYSTEM			0.041	-0.048	0.130	0.364	
c SEROTONINERGIC SYSTEM	Friedman 2005	Citalopram	-0.243	-0.868	0.382	0.447	-0.69
c SEROTONINERGIC SYSTEM	Sumiyoshi 2007	Buspirone	0.141	-0.363	0.646	0.583	0.66
c SEROTONINERGIC SYSTEM	Akhondzadeh 2009	Ondansetron	0.100	-0.597	0.797	0.778	0.36
c SEROTONINERGIC SYSTEM	Berk 2009	Mitazapine	0.140	-0.518	0.799	0.676	0.50
c SEROTONINERGIC SYSTEM	Piskulic 2009	Buspirone	-0.539	-1.436	0.359	0.239	-1.12
c SEROTONINERGIC SYSTEM	Sternberg 2010	Mitazapine	0.489	-0.151	1.130	0.134	1.59
c SEROTONINERGIC SYSTEM	Morozova 2012	Latrepirdine	-0.588	-1.202	0.026	0.061	-1.81
c SEROTONINERGIC SYSTEM	Niitsu 2012 50mg	Fluvoxamine	0.029	-0.533	0.592	0.919	0.20
c SEROTONINERGIC SYSTEM			-0.026	-0.262	0.209	0.827	
f GABA-ERGIC SYSTEM	Lewis 2008	MK-0777	0.769	-0.241	1.780	0.136	1.02
f GABA-ERGIC SYSTEM	Buchanan 2011 16mg	MK-0777	0.023	-0.625	0.671	0.944	-0.99
f GABA-ERGIC SYSTEM	Buchanan 2011 6mg	MK-0777	0.337	-0.345	1.019	0.333	0.19
f GABA-ERGIC SYSTEM			0.284	-0.162	0.730	0.212	
g NORADRENERGIC SYSTEM	Friedman 2001	Guanfacine	0.718	0.075	1.361	0.029	1.62
g NORADRENERGIC SYSTEM	Friedman 2008	Atomoxetine	-0.064	-1.019	0.891	0.896	-0.84
g NORADRENERGIC SYSTEM	Kelly 2009	Atomoxetine	-0.045	-0.852	0.763	0.914	-0.99
g NORADRENERGIC SYSTEM			0.307	-0.157	0.772	0.194	
h MISCELLANEOUS	Sevy 2005	Modafinil	0.789	-0.085	1.664	0.077	1.89
h MISCELLANEOUS	Goff 2008 100mg	Sildenafil	-0.213	-0.872	0.445	0.526	-0.45
h MISCELLANEOUS	Goff 2008 50mg	Sildenafil	-0.657	-1.332	0.017	0.056	-1.73
h MISCELLANEOUS	Kane 2010 150mg	Armodafinil	-0.102	-0.862	0.657	0.792	-0.11
h MISCELLANEOUS	Kane 2010 100mg	Armodafinil	-0.016	-0.748	0.716	0.966	0.12
h MISCELLANEOUS	Kane 2010 50mg	Armodafinil	-0.318	-1.055	0.419	0.398	-0.68
h MISCELLANEOUS	Bobo 2011	Armodafinil	-0.493	-1.009	0.022	0.061	-1.63
h MISCELLANEOUS	Javitt 2012 30mg	Davunetide	-0.008	-0.639	0.623	0.980	0.16
h MISCELLANEOUS	Javitt 2012 5mg	Davunetide	0.299	-0.344	0.943	0.362	1.08
h MISCELLANEOUS	Yi 2012	Rosiglitazone	-0.043	-0.903	0.818	0.923	0.04
h MISCELLANEOUS	Huerta-Ramos 2014	Raloxifene	0.250	-0.671	1.171	0.595	0.66
h MISCELLANEOUS	Lees 2017	Modafinil	0.183	-0.252	0.618	0.409	1.07
h MISCELLANEOUS			-0.059	-0.261	0.143	0.565	
Overall			0.060	-0.005	0.124	0.069	

Hedges's g and 95% CI

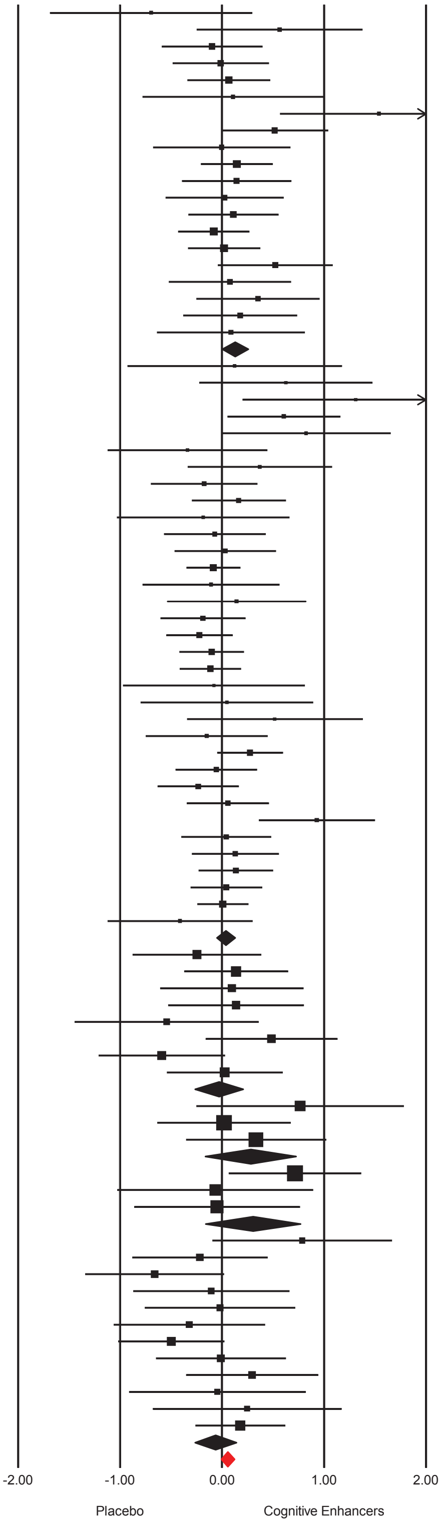

Supplement: Supplementary file 1 — Supplementary material [file 41537_2018_64_MOESM1_ESM.pdf]
